# Design and optimization of a novel loop configured MSF desalination system with reservoir tanks for minimal liquid discharge

**DOI:** 10.1038/s41598-025-18858-2

**Published:** 2025-10-09

**Authors:** Mahsa Khavari, Mohammad Akhlaghi, Nowrouz Mohammad Nouri

**Affiliations:** School of Mechanical Engineering, Iran University of Science and Technology, Tehran, Iran

**Keywords:** Small scale desalination, Multi-Stage-Flash, Multi-objective optimization, Minimum liquid discharge, Environmental sciences, Energy science and technology, Engineering

## Abstract

The present study proposes a novel environmentally friendly desalination system with loop-configured Multi-Stage Flash (MSF) system, suitable for small-scale applications. Some of the features that make the system’s initial concept attractive are: simplicity in design, operation and maintenance by eliminating the use of pipes in the condensers, minimum liquid discharge, which ultimately results in the highest possible water recovery; and the ability to integrate with low-temperature Heat sources which makes it a convenient option to apply in deserts and remote areas. Moreover, the system employs passive cooling through soil to enhance vapor condensation. In the present paper energy evaluation is carried out. Additionally, multi-objective optimization is used to minimize specific energy consumption and the number of water circulation cycles among condensation chambers before reaching a steady state. Subsequently, to determine the impact of key variables on system performance, a parametric study is performed. The results show that using the optimal decision variables, the proposed system can produce 4.7 L/h of fresh water with a water recovery ratio of 96% and a gain output ratio of 1. In addition, the corresponding specific energy consumption is 0.6307 kWh/L.

## Introduction

 Water supply for agriculture and drinking is a persistent problem in neglected and disadvantaged areas with little access to fresh, high-quality water supplies. Access to salty, low-quality water is a serious problem in many of these places, endangering not just human health but also local ecosystems and sustainable development. As a result, there is a great need for developing and improving technologies that can turn salty or poor-quality water into fresh water^1^.

One of the best ways to deal with the water shortage in remote areas is to install desalination units. These systems need to function well in challenging environments with limited and poor-quality water supplies. For this purpose, using technology with a high-water recovery potential becomes especially crucial. This feature guarantees that a comparatively small amount of contaminated or salty water can be converted into a larger volume of fresh water. Brine discharge, which can be harmful to the environment, is also an important concern in these systems^2^. Thus, it is essential to employ technologies that reduce brine waste while still achieving high water recovery.

The negative impacts of desalination systems on the environment can be reduced by employing a number of strategies. The first strategy is to power desalination plants with renewable energy sources, such as solar or wind^3^. In addition, according to research, solar desalination systems may be a good fit for disadvantaged areas since they can function well and use renewable energy to meet their energy needs, particularly in places with strong sun radiation. This subject has been the object of numerous studies. For example, the study conducted by Ganora^4^ showed that it is feasible to use photovoltaic (PV) energy to power large-scale reverse osmosis (RO) desalination plant in the Mediterranean region. It could supply desalinated water for almost 200 million people. Another study focused on how adding various energy storage materials might improve the performance of a conical solar distillation system^5^. Poredos et al.^6^ examined an 8-stage passive solar membrane distillation (MD) system enhanced with stage temperature boosting (STB), which directed low-level temperature Heating energy to the final stages condensers. The results indicated that STB-MD systems, particularly those with 16 stages, can be a better alternative to photovoltaic reverse osmosis (PV-RO) systems, improving the production of freshwater using low-temperature heat sources. The second strategy is to use minimal or zero liquid discharge (MLD or ZLD) approaches to treat and remove the brine generated by desalination plants^7,8^. Over 95% of the freshwater was recovered using the MLD and ZLD approaches. However, because ZLD and MLD are membrane and thermal-based processes, they are associated with high costs and energy consumption^9^. The third strategy is to use alternate desalination technologies, like membrane distillation or forward osmosis (FO), which need less energy and generate less brine than conventional desalination techniques^3^. Since FO is a naturally occurring osmosis-driven process, it seems to be a more energy-efficient solution (0.8–13 kWh/m^3^) and can recover up to 98% of freshwater^10^. Additionally, integrating several desalination methods can be another strategy to reduce the amount of brine produced during desalination processes^11^. For example, Zhang et al.^12^ studied a hybrid forward osmosis–membrane distillation (FO-MD) system for sustainable water recovery from oily wastewater using lab-fabricated hollow fiber membranes for both FO and MD. According to their results, the integrated FO-MD system successfully treated high-salinity oily wastewater, recovering over 90% of the water with minimal oil and salt residues. Regardless, the membrane-based methods are susceptible to fouling, need frequent maintenance and extensive pretreatment, and lead to difficulties when disposing of membrane material. Due to these limitations, thermal-based desalination processes continue to be used in some regions. For example, the multistage flash (MSF) desalination is the dominant desalination method among thermal-based methods in the Gulf Cooperation Council countries, due to its reliability and effectiveness in treating high-salinity seawater with minimum additional treatment requirements.

The MSF system has attracted significant interest over the past decades. Baig et al.^13^ studied the fouling factor of the MSF system in brine heaters. They discovered a close correlation between this fouling factor, the heat transfer coefficient, and the overall performance of the system. Shaaban et al.^14^ examined the integrated solar combined cycle to drive the MSF desalination unit. Their proposed cycle produced 16,300 m^3^ of fresh water per day and generated approximately 127 MW of electricity as well. When it comes to optimizing the MSF process commonly used to treat wastewater, researchers often examine it in terms of energy and exergy efficiency, environmental impact and economic aspects. Harandi et al.^15^ conducted an optimization of the efficiency of the multi-stage flash brine recirculation with thermal vapor compression (MSF-BR-TVC) system through genetic algorithms and examined different configurations of the MSF desalination system. Their findings indicated that for all configurations, increasing the number of stages in the heat recovery section raises the system’s performance coefficient. Additionally, it was discovered that the temperature of the last stage, the input steam temperature, and the top brine temperature (TBT) significantly affect the system performance coefficient. Finally, according to their results, the maximum gain output ratio (GOR) of 9.9 occurred in a system featuring two TVCs drawn from the intermediate stages. Sanaye and Asgari^16^ used the Pareto approach and 4E analysis to study how the MSF desalination plant could be integrated with the steam turbine cycle. The results revealed the ideal number of stages and the optimum TBT were 18 stages and 109.9 °C, respectively. In another research, Sellami et al.^17^ conducted optimization on the once-through MSF with TVC (MSF-OT/TVC) system using the Pareto method. Two important parameters were recognized according to their results: the first stage’s temperature and the system’s incoming water flow rate. In their study, maximizing the water production and minimizing the motive steam and electricity consumption were the objective functions. The optimization result gave a 23% reduction in the amount of inlet feed water into the system with a 7.3% reduction in motive steam consumption. Tayyeban et al.^18^ conducted a comparative study between the MSF and MSF-TVC systems for the desalination of wastewater from existing refineries while considering various heat losses intrinsic to the refinery processes. Additionally, optimization for both MSF and MSF-TVC systems was done on performance using the TOPSIS and the Pareto solution. It should be noted that parameters associated with energy, exergy, and economic issues such as the GOR, payback ratio, and exergy efficiency were all taken into account at the same time. Consequently, the MSF system’s optimal first-stage temperature (highest system temperature) and desalination stage number were 130 °C and 32 stages, while the MSF-TVC system’s optimal values were 115 °C and 25 stages. A number of published papers^19–23^ focused on the second-law analysis of the MSF method. For example, Almerri et al.^24^ tried to simulate an industrial MSF plant and later examined an exergy loss and process thermodynamic limitations. According to the results, the highest exergy loss was observed in the Heat recovery section, which was 55.5% of the total exergy loss. In another study, Farhadi et al.^25^ worked on the Abadan refinery’s MSF-BR system with the viewpoint of energy-exergy and exergoeconomics. They also studied the effect of TBT, number of stages, and temperature of the surroundings on the system efficiency. One of the results claimed that, as TBT increases, exergy efficiency, GOR, and distillate water production increase by 34%, 47%, and 47%, respectively.

The MSF method has drawbacks that make it challenging to apply in remote areas. For example, saltwater contaminants can cause scaling, fouling, and corrosion problems in the MSF system due to their accumulation on heat transfer surfaces, which can negatively affect its performance and need regular maintenance and cleaning^26^. Furthermore, MSF has a large material and land footprint with comparatively low recovery ratio^27^. The performance of the MSF process, particularly productivity and efficiency, has been enhanced by hybridizing MSF with several other technologies. Many studies investigated these hybrid systems^28,29^. For example, a hybrid MSF–humidification dehumidification (HDH) system was examined by Lawal^30^. The hybrid system improved production and performance while decreasing brine rejection, according to the results. The hybrid system achieved a water recovery ratio (RR) of 44.86 and a GOR of 8.73. However, while integrating different technologies, it is important to carefully consider the complex aspects of system design, operation, and maintenance. Additionally, desalination systems in rural places must be simple to use and maintain^31^. These regions usually suffer from a shortage of both technology and human resources. Therefore, improving the water supply and quality of life requires methods that are simple for local communities to use and don’t need extensive maintenance.

Thus far, certain small-scale solar desalination systems have been developed to provide freshwater in rural and off-grid regions, including solar stills that utilize direct solar heating and condensations; humidification–dehumidification (HDH), which uses air–water vapor exchange cycles; and thermoelectric (TE) systems that utilize the Peltier effect to create temperature gradients for water evaporation and condensation driving forces. These systems are, however, often challenged with applicability limitations: solar stills are often characterized with low water productivity^32^ HDH systems are indicative of complicated heat and mass transfer management^33^ and TE systems are open to poor energy efficiency and high cost per liter of yielded water^34,35^. These limitations hinder scalability and render these systems unsuitable for long-term implementation in remote communities.

The main objective of this research is to develop a novel, small-scale, eco-friendly desalination system that can be applied to meet the needs of isolated and arid areas. This desalination system, which utilizes a loop arrangement with two reservoir tanks to recover more than 95% of the water, has been inspired by the MSF process. Some of the paper’s highlights include:

- Providing a new small scale desalination system capable of using low-grade thermal energy sources with TBT of lower than 95 °C and working with flashing and condensation phenomena. Simple design, construction, and maintenance due to the lack of need for heat transfer tubes in condensing and flashing chambers, which results in low sensitivity to scale formation, makes it a suitable choice for use in wilderness and remote areas.
- Calculating the thermal and electrical energy consumption of the various system components by conducting an energy analysis of the proposed system.
- Performing a parametric study to determine the effects of various parameters on the system performance, such as feed water salinity and TBT.
- Developing a multi-objective optimization to identify the distribution pattern of decision variables, determine the appropriate range for each variable in the model, and present the Pareto Frontier of the model.
- Conducting a preliminary economic evaluation of the proposed system, and comparing its economic parameters and performance with those of other alternative small-scale desalination.

## System description

**Fig. 1:**
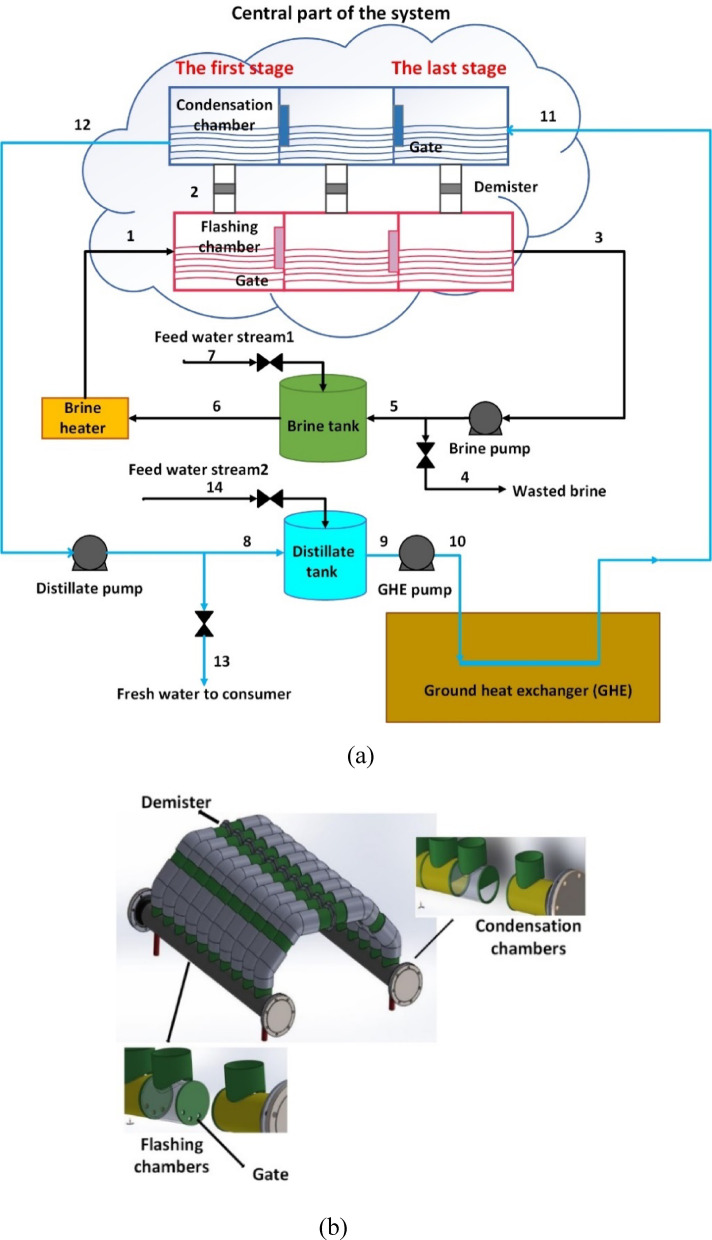
(**a**) Schematic diagram of the studied desalination system, drawn using Microsoft Visio 2021 (https://www.microsoft.com/visio); (**b**) Schematic of the central part of the desalination system, drawn using SolidWorks 2019 (https://www.solidworks.com).

The proposed desalination system in this paper works on the basis of flash evaporation and condensation. Its schematic diagram is shown in Fig. 1(a). The system consists of two main sections, namely: 1- the central section, 2- the complementary section. The complementary section includes two storage tanks for storing brine and desalinated water, two pumps, a brine heater and a water-cooling system. This system can be considered as a loop-configured MSF, as the working principle of the central section of this system is similar to MSF because it uses condensation and flashing phenomena to desalinate salt water but is loop-configured because water from central section is drawn into reservoir tanks by two suction pumps. In other words, water is recovered from the central section by the complementary section of the system.

The central part of this system as shown in Fig. 1(a) and 1(b), consists of a number of consecutive flashing and condensation chambers. As shown in Fig. 1(b), there is a connecting pipeline above each flashing chamber to the top of each condensation chamber to allow steam to pass through. These pipes are fitted with demister pads to remove liquid droplets and mist from vapor stream. The preheated brine (stream 1) enters the first flashing chamber. The pressure in this chamber is adjusted to be less than the saturation pressure at the temperature of stream 1. Consequently, upon entering the first flashing chamber, part of stream 1 is suddenly evaporated, and the vapor passes through a demister and enters the first condensation chamber (stream 2). Gradually, the pressure in the consecutive flashing chambers decreases to ensure a sudden flashing of a portion of the brine in each chamber, and finally, the concentrated brine is discharged from the last flashing chamber (stream 3). On the other hand, the saltwater stream (stream 11) enters the last condensation chamber, and the vapor flashed in the last flashing chamber is condensed Here due to its contact with salt water surface at lower temperature so that the water stream is directed to the adjacent condensing chamber. Finally, stream 12 is discharged from the first condensation chamber and directed to the distillate tank. The temperature of the water stream increases and its salinity decreases as it passes through the last condenser to the first condenser. It should be noted that the pressure in each condensing chamber is lower than the pressure in its corresponding flashing chamber. Thus, due to the existing pressure difference, the vapor can easily flow from the flashing chamber to the condensing chamber.

As mentioned earlier, the complementary section of the system uses two tanks for brine and distilled water at different heights compared to the central section. The flow is discharged from the chambers into their corresponding reservoir tanks by means of two pumps. The outlet of the brine tank (stream 6) receives the thermal energy in the brine heater to reach the required temperature. Alternatively, outlet of the distillate tank (stream 9) is passively cooled by the ground heat exchanger (GHE) to reach the temperature required for vapor condensation in all the condensing chambers. It should be mentioned that the Heater in the proposed desalination system can be driven by solar energy. In addition, GHE is able to cool the water through buried polyethylene pipes surrounded by soil, taking advantage of the fact that soil temperature at depths of 2 to 4 m remains nearly constant and is unaffected by seasonal variations^36,37^. Moreover, a circulation pump (GHE pump) is employed to overcome the pressure drop caused by frictional losses along the buried pipe.

As can be seen in Fig. 1(a), there are four main valves in this system. These valves are used to adjust the flow rate of feed water streams (streams 7 and 14), fresh water delivered to consumer known as the fresh water valve (stream 13) and rejected high-salinity brine known as the wasted brine valve (stream 4). When the system operation begins, both tanks are initially filled with feedwater, and then all valves are closed. The brine tank’s water gradually becomes saltier over time and its volume decreases, while the salinity of the water in the distillate tank decreases and its volume increases.

When the salinity of the water in the distillate tank reaches the desired and suitable salinity level of 100ppm in this study, the valve of the feed water stream 2 and the fresh water valve are opened so that the water in the distillate tank becomes salty again and it is recovered into the condensing chambers to desalinate. This process continues until the concentrated brine in the brine tank reaches a predetermined salinity level which in this study is assumed to be equal to 70000ppm, at which time the wasted brine valve and the valve of feed water stream1 are also opened. Then, the system enters a steady state operating phase with all valves open, and the flow rates of the corresponding streams (Streams 4, 7, 13, 14) can be adjusted to achieve continuous freshwater production with desired recovery ratio.

### Site description and environmental assumptions

This evaluation takes into consideration the climatic and environmental characteristics of Chabahar, a city in southeastern Iran (latitude 25.281° N and longitude 60.651° E), to ascertain the feasibility and efficiency of the proposed thermal desalination system. This region is characterized by high solar irradiation, humid and torrid coastal climate, and a scarcity of freshwater. Traditionally, rural people in this area utilize common surface water ponds called in the region “Hootag” as their major water source supply. Despite the initial water source usually originating from rainfalls or runoff, soil salinity, high evaporation rate, long storage duration, and inability to renew water often render the salinity content in these ponds higher than the acceptable limit for drinking water uses. Although the exact salinity of these water supplies varies spatially and seasonally, 3000 ppm concentration falls in their common range and therefore this concentration in this research is chosen to represent the typical range in evaluating system performance. Moreover, certain assumptions applied in the model, such as soil thermal conductivity, annual mean air temperature, and total solar radiation intensity, are all derived from conditions in this region^38^.

## Methods

The thermodynamic modeling of the proposed desalination system is carried out from energy perspective under steady state condition. On the other hand, the first law of thermodynamics is used in the model. Note that heat losses are assumed to be negligible.

### Thermodynamics examination

When operating in a steady state, the equation for mass balance can be considered Eq. (1)^39^.

1 $$\sum {{{\dot {m}}_{in}}=} \sum {{{\dot {m}}_{out}}}$$

Where $\:\dot{m}\:$is mass flow rate (kg/s). The *in* and *out* indexes represent the inlet and outlet streams of a control volume.

Salt balance equation can be considered as Eq. (2).

2 $$\sum {{{\dot {m}}_{in}}.{X_{in}}=} \sum {{{\dot {m}}_{out}}} .{X_{out}}$$

Where *X* represents the water salinity (ppm).

The general form of the energy balance equation can be expressed as Eq. (3)^39^, and is used for each component listed in Table 1.

3 $$\sum {{{\dot {Q}}_{in}}+} \sum {{{\dot {W}}_{in}}+} \sum {{{\dot {m}}_{in}}.{h_{in}}=} \sum {{{\dot {Q}}_{out}}+\sum {{{\dot {W}}_{out}}+} } \sum {{{\dot {m}}_{out}}} .{h_{out}}$$

Where $\:\dot{Q}$ is heat transfer rate (kW), $\:\dot{W}\:$represents the work performed (kW) and *h* is specific enthalpy (kJ/kg).

#### Flashing and condensation chambers

**Fig. 2:**
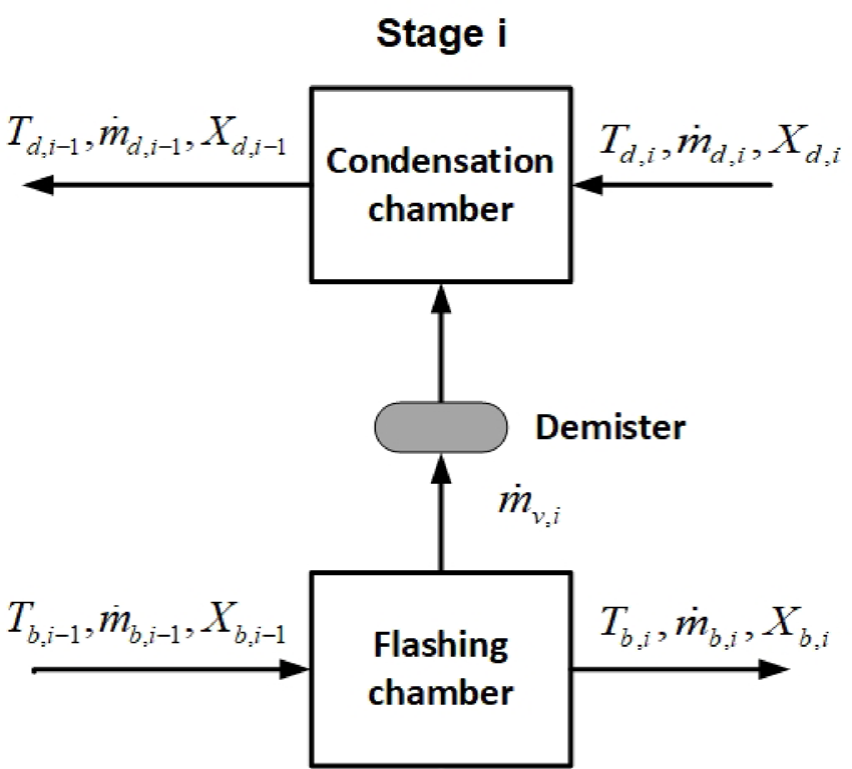
A top view of the i^th^ stage and its input and output streams.

In this section, the governing steady state equations of the system include mass, salinity and energy conservation equations are separately formulated for the i^th^ stage of the system The inlet and outlet flow of a specific stage (i^th^) are illustrated in Fig. 2. Each stage includes a flash chamber and a condensation chamber and a demister pad.

The mass balance equations for the i^th^ flashing and condensation chambers are written as Eq. (4) and Eq. (5), respectively.

4 $${\dot {m}_{b,i - 1}}={\dot {m}_{V,i}}+{\dot {m}_{b,i}}$$

5 $${\dot {m}_{d,i - 1}}={\dot {m}_{d,i}}+{\dot {m}_{V,i}}$$

Where $\:{\dot{m}}_{v,i}$ is the mass flow rate of vapor flashed in the i^th^ flashing chamber. $\:{\dot{m}}_{b}\:$and $\:{\dot{m}}_{d}$ are mass flow rate of brine and distillated water, respectively. As shown in Fig. 2, index *i* refers to the output stream of the i^th^ flash box and the inlet stream of the i^th^ condensation box, while index *i-1* relates to the inlet stream of the i^th^ flash box and the output stream of the i^th^ condenser box.

Equation (6) and Eq. (7) express the salt mass balance for flashing and condensation chamber i^th^ respectively.

 

6 $${X_{b,i - 1}}.{\dot {m}_{b,i - 1}}={X_{b,i}}.{\dot {m}_{b,i}}$$

 

7 $${X_{d,i - 1}}.{\dot {m}_{d,i - 1}}={X_{d,i}}.{\dot {m}_{d,i}}$$

Where $\:{X}_{b}$ and $\:{X}_{d}$ represent the salinity of brine and distillated water, respectively.

Energy conservation equation for flashing and condensation chamber i^th^ is written as Eq. (8) and Eq. (9), respectively.

8 $${\dot {m}_{b,i - 1}}.{c_p}.({T_{b,i - 1}} - {T_{b,i}})={\dot {m}_{V,i}}.{\lambda _{V,i}}$$

9 $${\dot {m}_{d,i}}.{c_p}.({T_{d,i - 1}} - {T_{d,i}})={\dot {m}_{V,i}}.{\lambda _{V,i}}$$

Where $\:{T}_{b}$ and $\:{T}_{b}$ are the temperature of brine and distillated water, respectively (°C). Also, $\:{C}_{p}\:$is the specific heat (kJ. kg^-1^. °C^-1^) and $\:{\lambda\:}_{V}$ is latent heat of water evaporation (kJ/kg). $\:{C}_{p}$ and $\:{\lambda\:}_{V}$ are calculated based on the given correlations by El-Dessouky^40^.

#### Brine and distillate tanks

Mass conservation of brine and distillate tanks can be expressed as Eq. (10) and Eq. (11), respectively.

10 $${\dot {m}_5}+{\dot {m}_7}={\dot {m}_6}$$

11 $${\dot {m}_8}+{\dot {m}_{14}}={\dot {m}_9}$$

Salt mass balance equation for brine and distillate tanks can be written as Eq. (12) and Eq. (13), respectively:

12 $${\dot {m}_5}.{X_5}+{\dot {m}_7}.{X_7}={\dot {m}_6}.{X_6}$$

13 $${\dot {m}_8}.{X_8}+{\dot {m}_{14}}.{X_{14}}={\dot {m}_9}.{X_9}$$

Equation (14) and Eq. (15) represent the energy conservation of brine and distillate tanks, respectively.

14 $${\dot {m}_5}.{h_5}+{\dot {m}_7}.{h_7}={\dot {m}_6}.{h_6}$$

15 $${\dot {m}_8}.{h_8}+{\dot {m}_{14}}.{h_{14}}={\dot {m}_9}.{h_9}$$

Where *h* is specific enthalpy (kJ/kg).

Energy balance equation is given in Table 1 for the other components of the complementary section. It should be noted that to calculate the specific enthalpy of salt water the given correlations by Homig is used^41^.

#### Underground heat exchanger

To evaluate the thermal performance of the ground heat exchanger, the rate of heat exchange ($\:{Q}_{GHE}$) is calculated using the following Eq^42^..

16 $${Q_{GHE}}={\dot {m}_{GHE}}{C_p}({T_{in,GHE}} - {T_{out,GHE}})$$

Where $\:{T}_{in,GHE}$ and $\:{T}_{out,GHE}$ are the underground heat exchanger inlet and outlet temperatures, respectively. Alternatively, the heat exchange can be expressed in terms of the overall heat transfer coefficient (*U*) between buried pipe and soil, as follows:

17 $${Q_{GHE}}=UA\Delta {T_{LM}}$$

Where *U* is the overall heat transfer coefficient (W.m^−2^. °C^−1^), *A* is the heat transfer area (m^2^ and $\:{\varDelta\:T}_{LM}$ is the logarithmic mean temperature difference (*LMTD*), calculated using the Eq. (18)^42^:

18 $$\Delta {T_{LM}}=\frac{{({T_{in,GHE}} - {T_{out,GHE}})}}{{Ln(\frac{{{T_{in,GHE}} - {T_s}}}{{{T_{out,GHE}} - {T_s}}})}}$$

Where $\:{T}_{s}$ is the soil temperature which is approximated by the mean annual ambient air temperature, following ASHRAE recommendations^43^.

To estimate the overall heat transfer coefficient times area (*UA*) of the underground heat exchanger, a thermal resistance model is employed. The total resistance is treated as the sum of three main components in series: (i) the convective resistance inside the pipe due to the flowing fluid ($\:{R}_{water}$), (ii) the conductive resistance through the pipe wall ($\:{R}_{pipe}$), and (iii) the conductive resistance of the surrounding soil ($\:{R}_{soil}$). The overall heat transfer coefficient is then calculated by Eq. (19):

19 $$\frac{1}{{UA}}={R_{water}}+{R_{pipe}}+{R_{soil}}$$

$\:{R}_{water}$, $\:{R}_{pipe}$ and $\:{R}_{soil}$ are calculated using Eq. (20) to Eq. (22)^43^.

20 $$\left\{ \begin{gathered} {R_{water}}=\frac{1}{{{h_w}.{A_{in}}}} \hfill \\ {h_w}=\frac{{N{u_D}.{K_w}}}{D} \hfill \\ \end{gathered} \right.$$

Where $\:{h}_{w}$ is the internal convective heat transfer coefficient (W.m^-2^. °C^-1^). *A*_*in*_ is the internal heat transfer surface area of the pipe (m^2^. *Nu*_*D*_, *K*_*w*_ and *D* are Nusselt number, the thermal conductivity of the fluid (W.m^-1^. °C^-1^) and inner diameter of buried pipe, respectively.

21 $${R_{soil}}=\frac{{\ln ({\raise0.7ex\hbox{${2d}$} \!\mathord{\left/ {\vphantom {{2d} {{r_o}}}}\right.\kern-0pt}\!\lower0.7ex\hbox{${{r_o}}$}})}}{{2\pi .{K_s}}}$$

22 $${R_{pipe}}=\frac{{\ln ({\raise0.7ex\hbox{${{r_o}}$} \!\mathord{\left/ {\vphantom {{{r_o}} {{r_i}}}}\right.\kern-0pt}\!\lower0.7ex\hbox{${{r_i}}$}})}}{{2\pi .{K_p}}}$$

Where *r*_*o*_, *r*_*i*_ and *d* are the outer radius of pipe (m), the inner radius of pipe and the burial depth to centerline of pipe (m), respectively. *K*_*s*_ and *K*_*p*_ are the thermal conductivity of soil and pipe, respectively (W/m. °C). The outlet temperature of GHE predicted by the present model (Eq. 16 to 22) with a straight pipe assumption shows only a 2.1% deviation from the results of another reference^44^ which used a slinky-type ground heat exchanger, indicating good agreement and validating the applied methodology. Table 2 summarizes the characteristics of the GHE and the surrounding soil.

**Table 1:** The first law of thermodynamics expression for the components of the system complementary section.

Component	Equation of energy balance
Brine pump	$\:{\dot{m}}_{3}\left({h}_{5}-{h}_{3}\right)={\dot{W}}_{Brinepump}$
Distillate pump	$\:{\dot{m}}_{12}\left({h}_{8}-{h}_{12}\right)={\dot{W}}_{Distillatepump}$
Brine heater	$\:{\dot{Q}}_{heater}={\dot{m}}_{6}({h}_{1}-{h}_{6})$
GHE pump	$\:{\dot{m}}_{9}\left({h}_{10}-{h}_{9}\right)={\dot{W}}_{GHEpump}$

**Table 2:** The characteristics of the GHE and the surrounding soil^38,43^.

Parameter	Value
GHE pipe material	Polyethylene
Length of pipe (m)	55
Internal diameter (m)	0.004
External diameter (m)	0.006
Piping depth (m)	2
GHE circulating water mass flow rate (kg/h)	72
Soil temperature (°C)	27.7
Soil thermal conductivity (W/m/K)	1.87

#### Performance evaluation

Three parameters are considered to estimate the performance of the proposed desalination system: 1- Specific energy consumption (SEC), 2- Recovery ratio (RR) and 3- Gain output ratio (GOR).

The specific energy consumption of the system is defined as the total energy consumption of the system per mass unit of fresh water produced and is computed according to Eq. (23).

23 $$SEC=\frac{{\left( {{{\dot {Q}}_{heater}}+{{\dot {W}}_{Brinepump}}+{{\dot {W}}_{Distillatepump}}+{{\dot {W}}_{GHEpump}}} \right)}}{{{{\dot {m}}_{13}}}}$$

Where SEC is the ratio of input energy to the fresh water production rate (kWh/L). In the above relation, the total energy consumed by the system includes the energy consumption of the heater ($\:{\dot{Q}}_{heater}$), and the pumps ($\:{\dot{W}}_{Brinepump}+{\dot{W}}_{Distillatepump}+{\dot{W}}_{GHEpump}$).

The system recovery ratio (RR) is defined as the mass ratio of the produced fresh water to the total saline feed water and calculated as Eq. (24).

24 $$RR=\frac{{{{\dot {m}}_{freshwater}}}}{{\sum {{\dot {m}}_{feedwater}}}}=\frac{{{{\dot {m}}_{13}}}}{{{{\dot {m}}_{14}}+{{\dot {m}}_7}}}$$

The third parameter discussed in this study for evaluating system performance is the Gained Output Ratio (GOR). In conventional desalination systems where the brine heater utilizes motive steam to heat the feedwater, GOR is defined as the ratio of the mass of produced freshwater to the mass of consumed steam. However, in the present study—since a direct thermal energy heater is used and no steam is injected for water heating—GOR is defined according to the references^45^ based on Eq. (25).

25 $$GOR=\frac{{{{\dot {m}}_{freshwater}}.{h_{fg}}}}{{{{\dot {Q}}_{heater}}}}$$

Where $\:{h}_{fg}$ is the latent Heat of vaporization of water at 100 °C (kJ/kg) and $\:{\dot{m}}_{freshwater}$ is equal to $\:{\dot{m}}_{13}$.

#### Optimization

Optimization of energy systems, especially in desalination, is an important area of ​​research aimed at improving the efficiency and sustainability of water treatment plants. This paper focuses on system optimization by considering two main objective functions. Both the specific energy consumption (SEC) and the number of cycles required for saline water to circulate in the condensers until reaching the steady state phase are simultaneously minimized in the optimization process. As mentioned earlier, the salt water must be constantly circulated through the system to reach the desired salinity in both reservoir tanks from the start of system operation until steady state operation phase. Therefore, the number of water circulation loops in the circuit, including the distillate tank and condensers is considered as a parameter that is relatively proportional to the time required for system initialization. Therefore, both objective functions need to be optimized concurrently through a genetic algorithm for multi-objective optimization while adhering to certain sensible constraints.

The major decision variables for the optimization are 1- heater outlet temperature ($\:{T}_{1}$), 2- flashing chambers outlet temperature ($\:{T}_{3}$) and 3- the ratio of brine tank outlet mass flowrate to the distillate tank outlet mass flowrate ($\:{\dot{m}}_{6}/{\dot{m}}_{9}$).

Table 3 lists the maximum and minimum limits for the decision variables. These constraints are determined by functional and physical constraints. For example, the upper bound of $\:{T}_{1}$ is determined based on the system’s operational requirements at low temperatures below the boiling point of water and the selection of the bottom limit functions to achieve effective flashing of vapors and is related to the minimum desired temperature difference of 10 °C between the primary and end flashing chamber.

Unlike classical MSF systems—in which the end flashing stage outlet temperature is conventionally in the range of 40°C^40,46^—the current study assumes a higher range of 65–70 °C for $\:{T}_{3}$ in optimizing the process. This choice attempts to maintain the last stage pressure in the range of 30 kPa, thereby reducing the necessary vacuum level to 19–25% below that obtained in classical MSF systems. Moreover, the flow ratio rate is restricted to a maximum of 1.3, owing to the observation that increased values will create a deficient temperature gap between the flashed vapor and the cold water in the condensation chambers. Alternatively, the ratio should not be reduced below 0.3, owing to the observation that lower values will significantly expand the number of circulation loops to attain steady state.

**Table 3:** The decision variables permissible lower and upper bounds.

Decision variables	Lower bound	Upper bound
$\:{T}_{1}$	80 °C	95 °C
$\:{T}_{3}$	65 °C	70 °C
$\:{\dot{m}}_{6}/{\dot{m}}_{9}$	0.3	1.3

### Validation

**Fig. 3:**
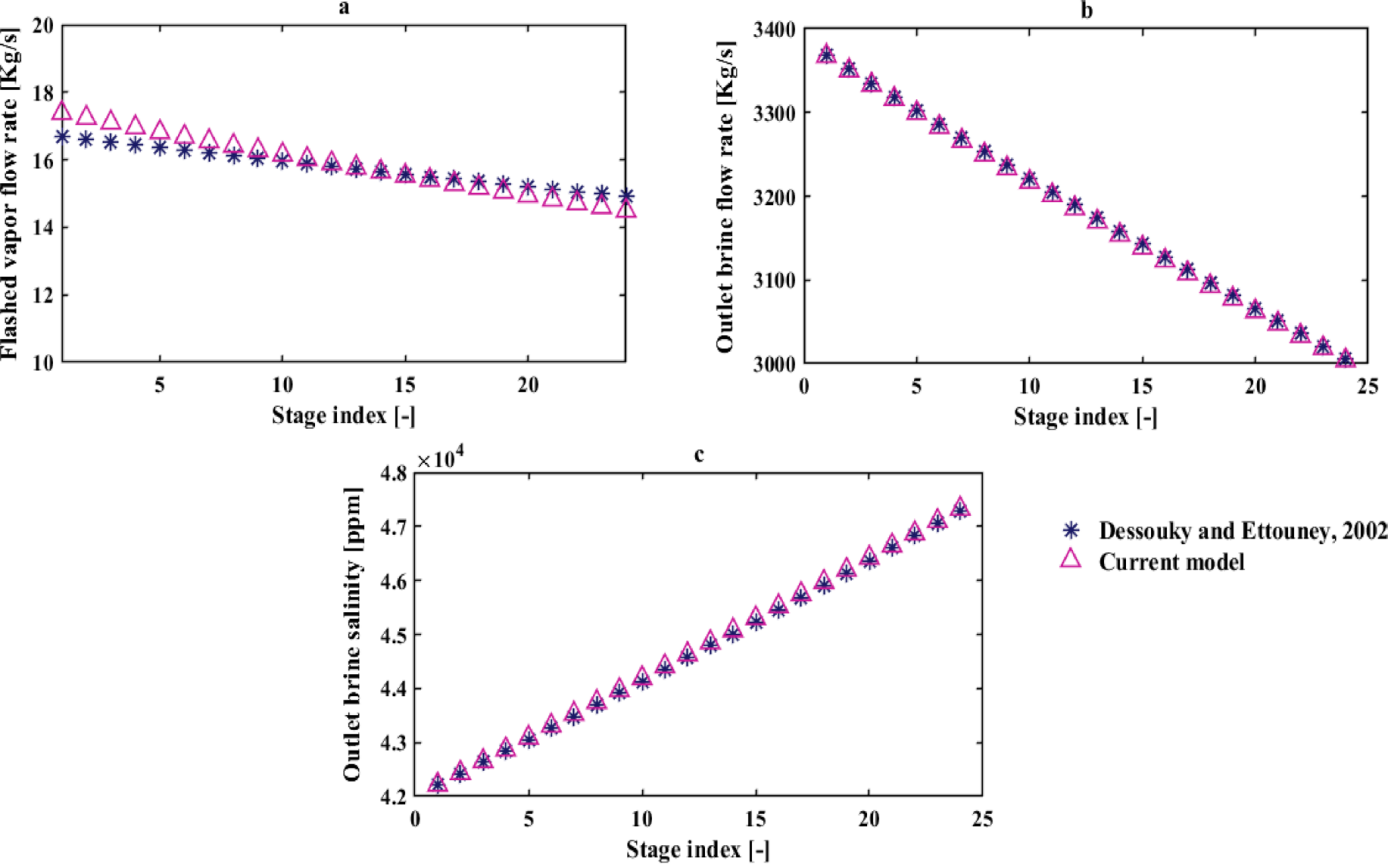
A stage-wise comparison between the results of El-Dessouky^40^ and the present study for: (**a**) Flashed vapor flowrate, (**b**) Outlet brine flow rate, (**c**) Outlet brine salinity.

Considering the novelty of the present desalination system, validation is solely performed for the flashing chambers assembly which is similar to the MSF-OT desalination system. The flashing chambers model’s validation is conducted against an analytical model developed by El-Dessouky (case study 6.4.3)^40^ for the MSF-OT system, comprised of 24 stages with TBT of 106 °C, brine reject temperature of 40 °C, feed water salinity of 42,000 ppm and an incoming brine flow rate to the first stage of 3384.8 kg/s. The results obtained from the analytical model and the current model in this paper for the salinity and mass flow rate of the brine stream exiting each flashing chamber and the vapor flow rate flashed in each flash chamber are illustrated in Fig. 3. It is noted that the results presented for all three examined parameters exhibit very good agreement with the Dessouky model. Specifically, for different stages, the average deviation of both salinity and flow rate of the brine output from each stage from the Dessouky model is 0.11%, and the average deviation of the flashed vapor flow rate in each stage from the Dessouky model is 1.78%.

## Results and discussion

In this section, firstly, the results of multi-objective optimization for the studied desalination system are first presented. Then, the comprehensive parametric study is conducted to investigate the impact of various operational parameters on the system performance. Finally, the results of the economic assessment are provided, followed by a technical and performance comparison between the proposed system and other existing desalination technologies.

### Optimization

**Fig. 4:**
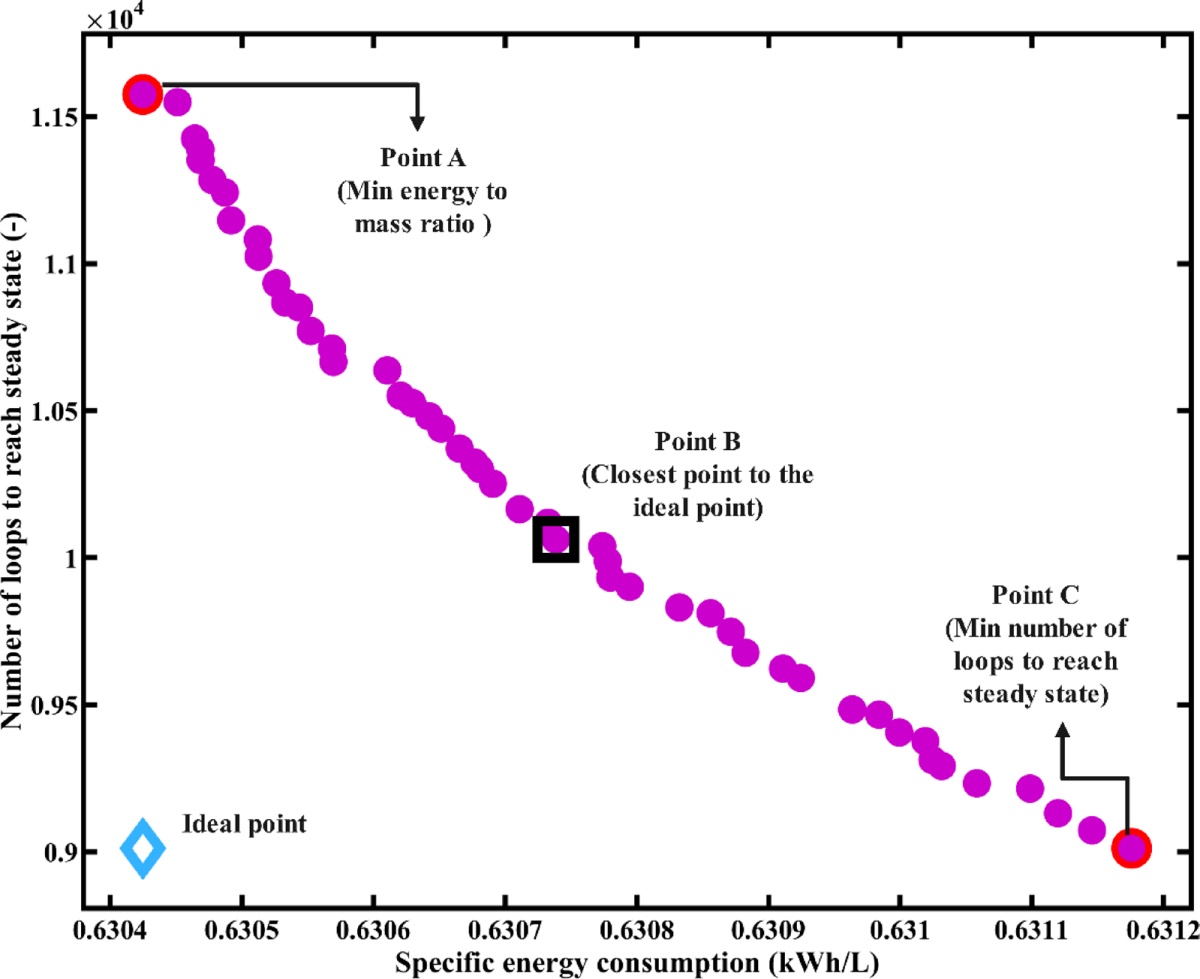
The studied model’s Pareto Frontier.

Figure 4 shows the suggested model’s Pareto Frontier. The findings indicate that the suggested model can achieve the minimum SEC of 0. 6304 kWh/L with the maximum number of loops before reaching the steady state of 11,577 (Point A), the closest point on the Pareto to the ideal point (optimal solution within the non-dimensional Pareto) with the SEC of 0. 6307 kWh/L and number of loops of 10,071 (Point B) and the minimum number of loops of 9005 (Point C).

Table 4 gives the values of decision variables and objective functions for three points (A, B, and C) on the Pareto Frontier. Based on the data presented in this table, the ideal heater outlet temperature ($\:{T}_{1}$) is around the 88 °C for the point with minimum SEC (Point A). This variable increases to 91.5 °C at the point closest to the ideal point (Point B), and reaches a much higher value near the upper bound of 95 °C at the point corresponding to the minimum number of loops required before reaching steady state (Point C).Flashing chambers outlet temperature ($\:{T}_{3}$) has the optimum value of about 65 °C for all points (A, B, C). In addition, the ratio of the brine tank outlet mass flowrate to the distillate tank outlet mass flowrate ($\:{\dot{m}}_{6}$/$\:{\dot{m}}_{9}$) remains close to its upper bound of 1.3 for all selected Pareto points (A, B, and C).

It is observed from the Pareto frontier (Fig. 4) that the variation in SEC is extremely limited across all optimal solutions. This limited variation in SEC across the Pareto solutions suggests that, within the defined bounds and configuration, the system’s energy efficiency remains relatively unaffected by the selected decision variables. Therefore, the optimization primarily affects the startup time objective.

**Fig. 5:**
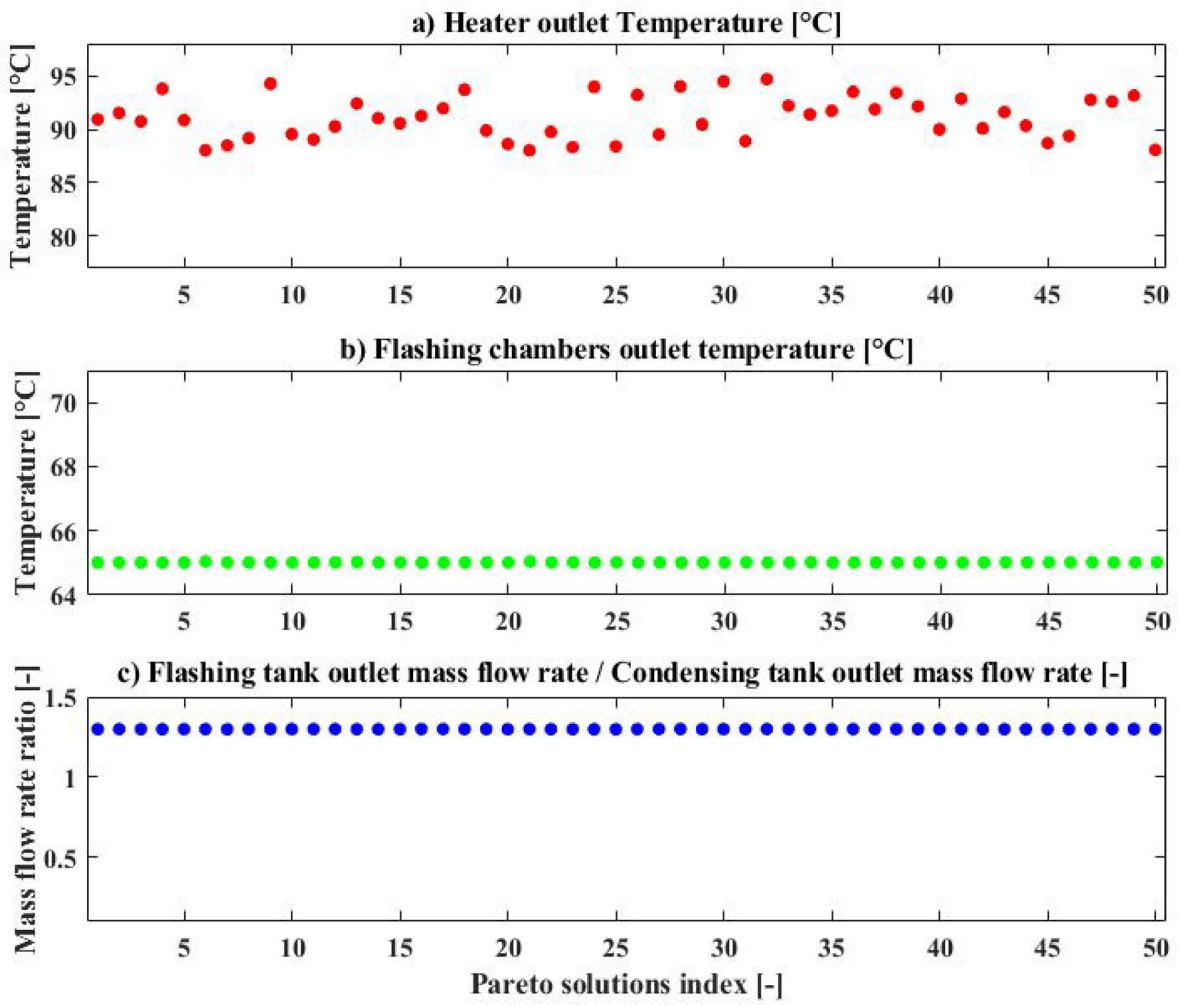
Scatter of the distribution for (**a**) heater outlet temperature, (**b**) flashing chambers outlet temperature, (**c**) the ratio of brine tank outlet mass flow rate to the distillate tank outlet mass flow rate for proposed model.

Figure 5 shows scatter plots of the distribution of decision variables. As shown in Fig. 5(a), the heater outlet temperature ($\:{T}_{1}$) exhibits a noticeable variation among solutions, indicating its significant influence on the trade-off between SEC and number of required loops. It can be seen that the optimal range for the heater outlet temperature (*T*_*1*_) lies approximately between 88 °C and 95 °C. In contrast, both the flashing chamber outlet temperature ($\:{T}_{3}$), shown in Fig. 5(b), and the mass flowrate ratio ($\:{\dot{m}}_{6}$/$\:{\dot{m}}_{9}$), shown in Fig. 5(c), remain almost constant across all Pareto points, with values close to 65 °C and 1.3, respectively. This consistency suggests that, within the defined bounds of the variables, the optimizer repeatedly selects these values as near-optimal. It also indicates that variations in system performance are predominantly governed by changes in $\:{T}_{1}$.

According to the Pareto frontier analysis, Point B was identified as the closest point to the ideal solution. While Points A, B, and C exhibit nearly identical specific energy consumption values (ranging between 0.6304 and 0.6312 kWh/L), they differ significantly in the number of loops required to reach steady state and in their decision variable $\:{T}_{1}$. Point C, which achieves the shortest startup duration, may appear superior at first glance. Nevertheless, it operates at the maximum $\:{T}_{1}$ (94.7 °C), which would likely cause lower operational stability or thermal stress in long-term operation. Point B, in comparison, attains comparable performance but with reduced $\:{T}_{1}$ (91.5 °C), with merely up to 10% extended startup time with respect to Point C. From a long-term operating consideration, that lower temperature will likely improve system reliability, particularly if operating with solar thermal energy. Depending on the specific design priorities, either Point B or C could be considered the best solution. However, in this study, due to a greater emphasis on operational stability, Point B was selected for further parametric analysis.

**Table 4:** The data related to Pareto frontier points’ decision variables and objective function values.

Point	Description	Decision Variables			Objective Functions	
		$\:{\varvec{T}}_{1}$	$\:{\varvec{T}}_{3}$	$\:{\dot{\varvec{m}}}_{6}/{\dot{\varvec{m}}}_{9}$	Specific energy consumption (SEC) [kWh/L]	Number of loops to reach steady state
A	Min energy to mass ratio	88.03	65.0434	1.2994	0. 6304	11,577
B	Closest point to the ideal point (Best point)	91.5	65.0123	1.2998	0.6307	10,071
C	Min number of loops to reach steady state	94.7	65.0117	1.3	0. 6312	9005

**Fig. 6:**
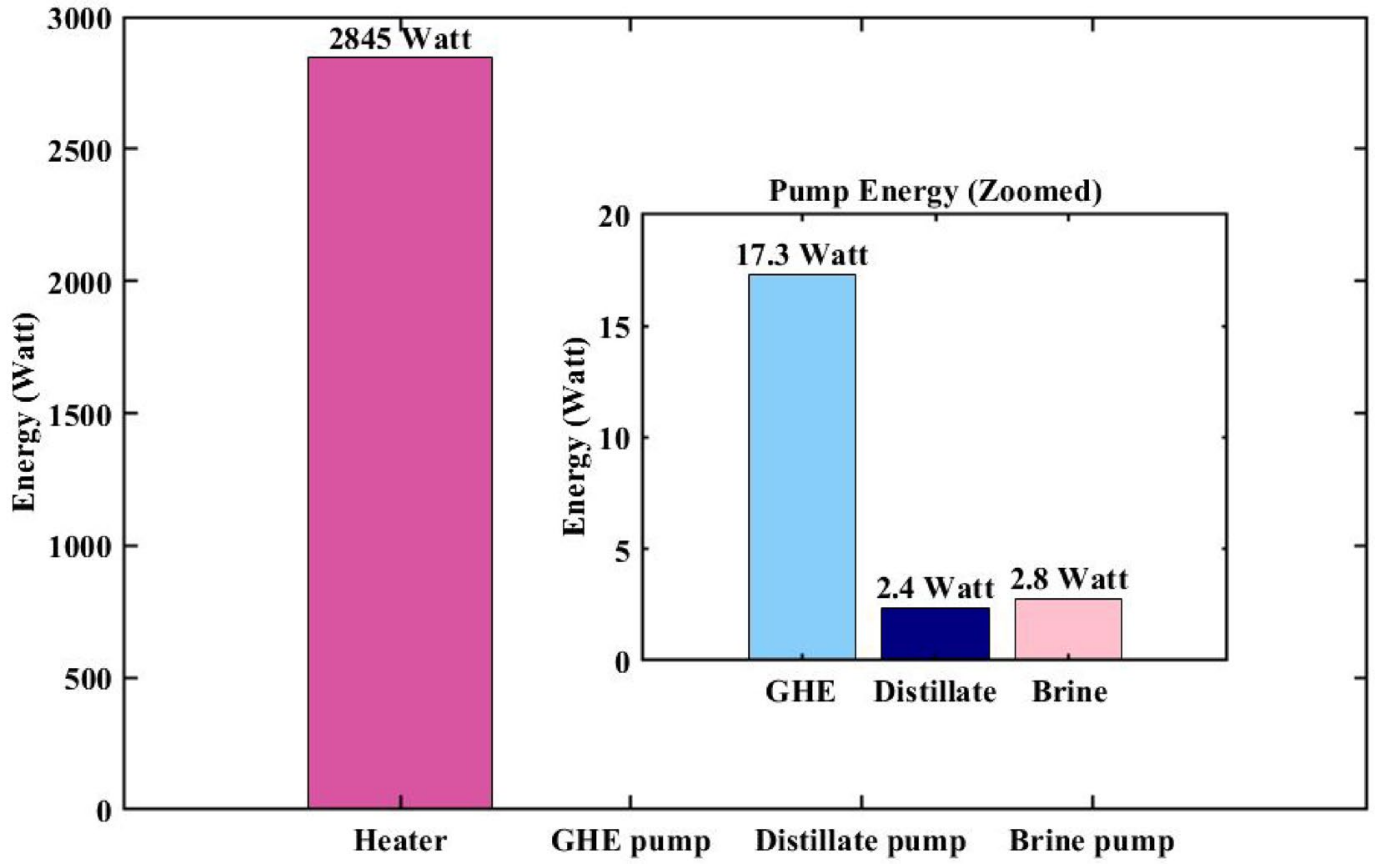
Energy input requirements of system components at the best point (Point B) on the Pareto frontier.

Figure 6 illustrates the required input energy to the system, broken down by component for point B on Pareto. As shown, the Heater constitutes the dominant share of the total energy demand, consuming approximately 2845 W. In contrast, the energy consumption of the pumps is negligible. Specifically, the GHE pump, distillate pump, and brine pump consume about 17.3 W, 2.4 W, and 2.8 W, respectively. Based on the energy consumption of heater and pumps shown in the Fig. 6 and a freshwater production rate of 4.7 L/h at point B on the Pareto frontier, the thermal SEC is 0.626 kWh/L, while the electrical SEC is 0.0048 kWh/L. In addition, thermal and saline properties of the key states of the complementary section at the Point B on the Pareto frontier are presented in Table 5.

**Table 5:** Thermal and saline properties of the key States of the complementary section at the best point (Point B) on the Pareto frontier.

State	Stream description	Temperature (T) [K]	Salinity (X) [ppm]	Specific enthalpy (h) [kJ/kg]
1	Heater outlet	364.65	70,177	350.37
3	The last flashing chamber’s outlet	338.16	73,637	246.78
4	Wasted brine	338.19	73,637	246.89
5	Brine tank inlet	338.19	73,637	246.89
6	Brine tank outlet	336.34	70,177	240.93
7	Feed water 1	303.15	3000	125.3
8	Distillate tank inlet	347.45	94.278	311.06
9	Distillate tank outlet	347.44	100.037	311.03
10	GHE inlet	347.65	100.037	311.89
11	GHE outlet	320.66	100.037	198.99
12	The first condensation chamber’s outlet	347.42	94.278	310.95
13	Fresh water to consumer	347.45	94.278	311.06
14	Feed water 2	303.15	3000	125.3

### Parametric study

This section focuses on how each system parameter and variable affects the system’s performance. For the purpose of conducting a parametric study, Point B on the Pareto frontier is selected as the reference point. This means that the decision variables corresponding to this point, as listed in Table 4, are considered as the system’s initial settings. In addition, the salinity of the feed water, minimum desired water salinity in the brine tank, and maximum desired water salinity in the distillate tank are assumed to be 3000 ppm, 70,000 ppm and 100 ppm, respectively. Then, with these parameters fixed, each variable or parameter in turn is varied to observe its impact on the system performance.

#### Heater outlet temperature

**Fig. 7:**
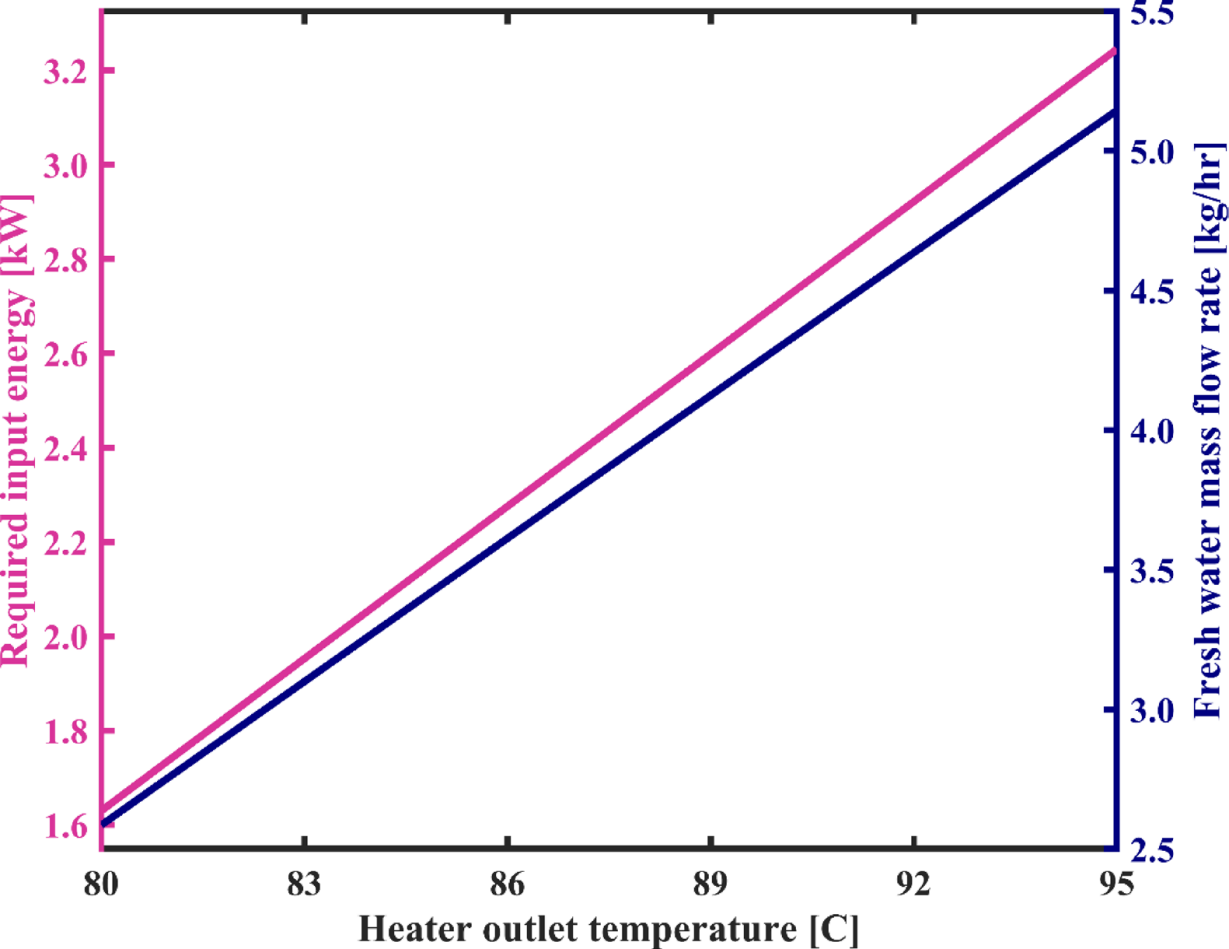
The variation of total energy consumption of the system and fresh water production capacity as a function of heater outlet temperature.

**Fig. 8:**
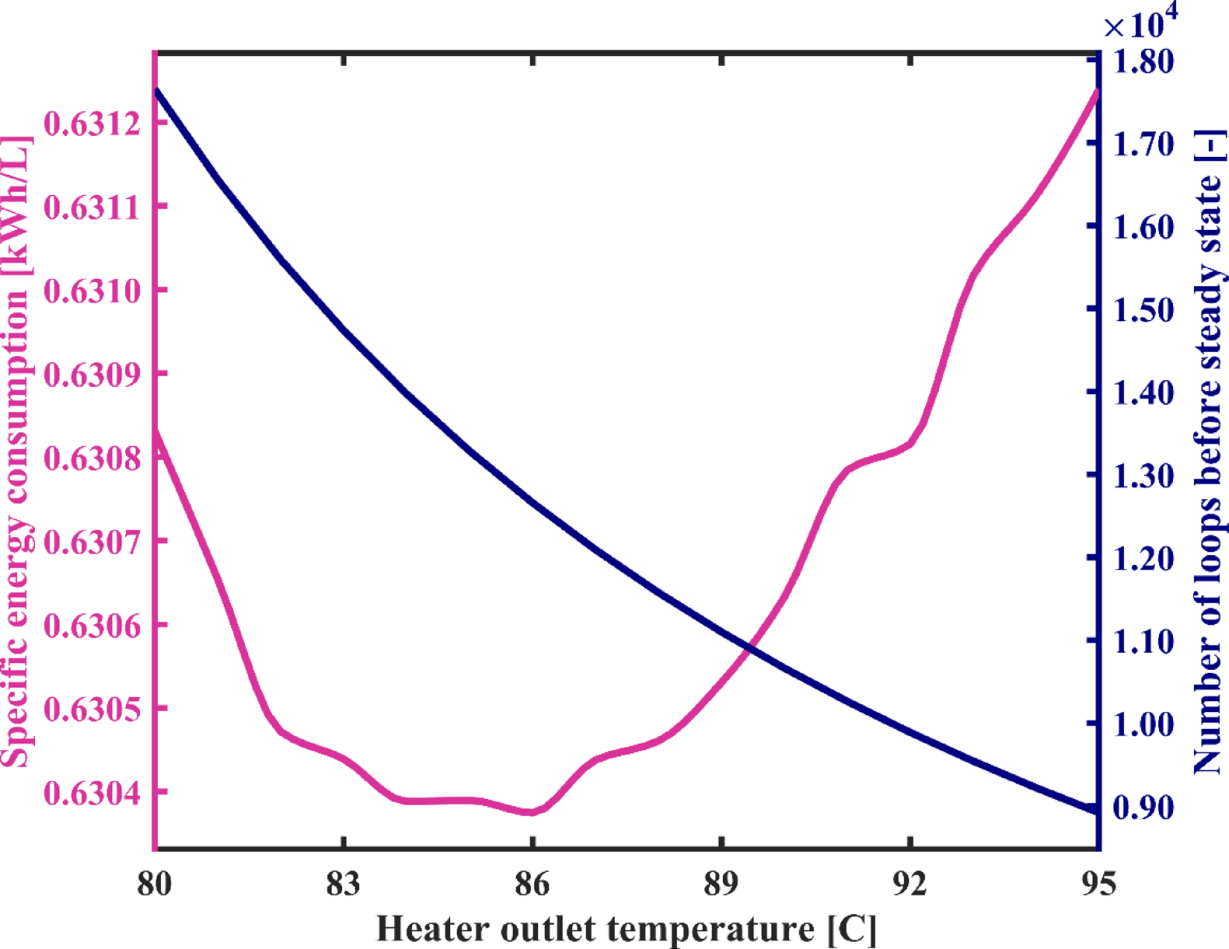
System’s specific energy consumption and number of water recirculation among condensation chambers before reaching steady state with varying heater temperature.

Figure 7 illustrates the variations in the consumption rate of energy and the flow rate of produced fresh water as a function of brine temperature at the outlet of the heater, while Fig. 8 depicts changes in the specific energy consumption (SEC) and the number of recirculating loops in the condensation chambers during system’s startup as a function of TBT or heater outlet temperature. In Fig. 7, it is noted that with an increase in Heater temperature from 80 °C to 95 °C, the energy consumption rate of the system increases from 1.63 kW to 3.24 kW. This is because, by maintaining the flashing chambers outlet temperature constant, brine heater requires more energy to provide this higher TBT. Consequently, the heating load of the heater increases. On the other hand, it is evident that with an increase in the TBT, more energy is introduced into the flashing chambers, resulting in an increase in the energy difference between the input and output of this assembly. Therefore, a greater amount of vapor is flashed at a higher temperature and directed to the condensation chambers. As a result, with an increase in the heater temperature, in each pass of water through the condensation chambers, the flow rate and temperature of water at the outlet of the condensation chambers increase, and its salinity decreases. This leads to three consequences. Firstly, due to the decrease in the salinity of the water output from the condenser assembly, the brackish water desalinated faster and reaches the desired acceptable salinity for fresh water sooner. In other words, the startup phase of the system is shortened, and the number of water recirculation before reaching a steady-state operation is significantly reduced. As shown in Fig. 8, with an increase in Heater temperature from 80 °C to 95 °C, the number of loops decreases from 17,646 to 8918. Secondly, due to the increase in the flow rate of water at the outlet of the condenser chambers, the flow rate of produced fresh water increases significantly. Thirdly, as the temperature of water leaving the condensation chambers increases, warmer water enters the ground heat exchanger (GHE), which in turn raises the temperature of the fluid exiting the GHE, due to the constant thermal properties of the heat exchanger and the surrounding soil. As a result, the entire set of condensers and flashing chambers operates at a higher temperature.

In Fig. 7, it is observed that the rates of increase in both the required energy and the freshwater production with rising heater temperature are approximately the same. Consequently, Fig. 8 shows that the SEC remains nearly constant, with very minor variations—around the fourth decimal place—which can be considered negligible.

#### Flashing chambers outlet temperature

**Fig. 9:**
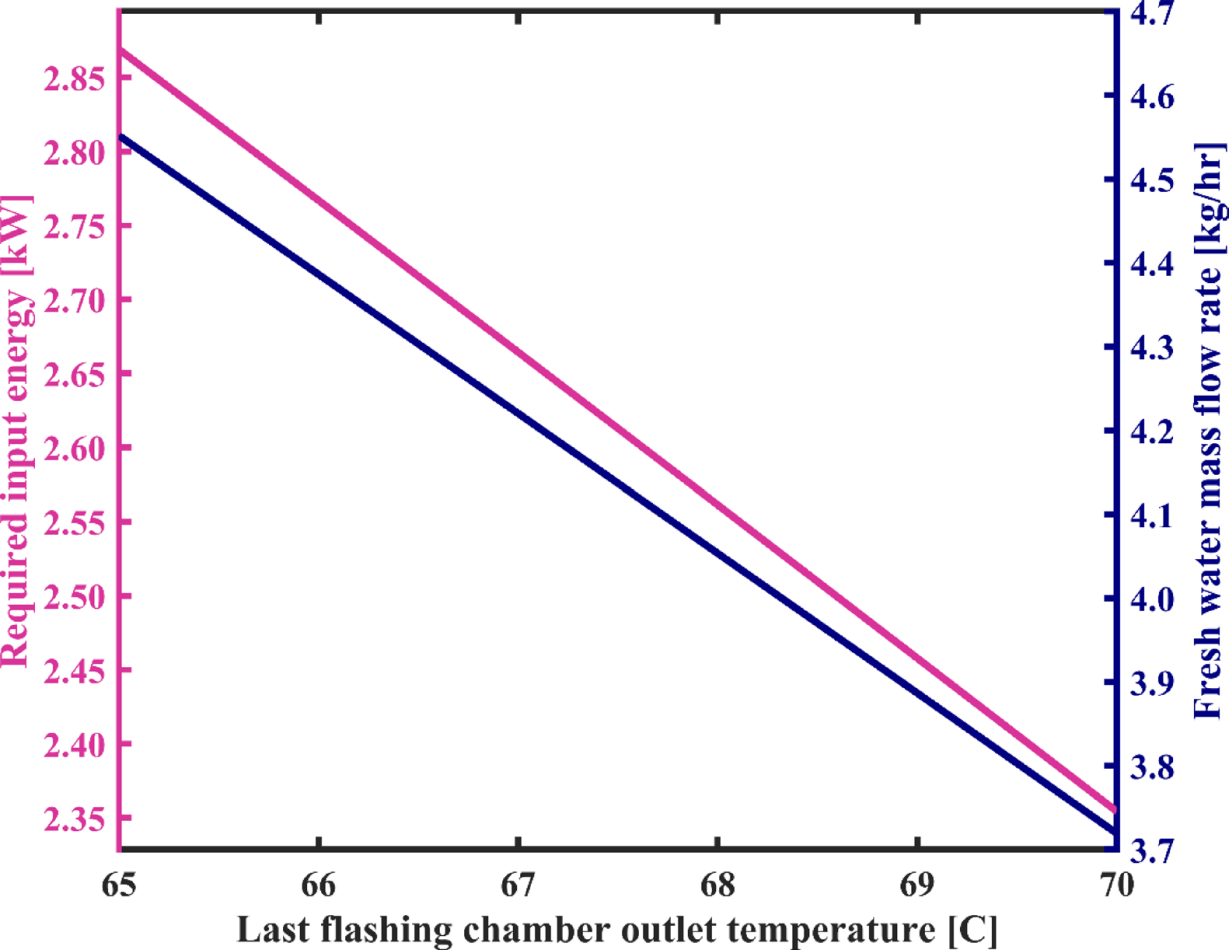
The total energy consumption of the system and fresh water production capacity vs. flashing chambers outlet temperature.

**Fig. 10:**
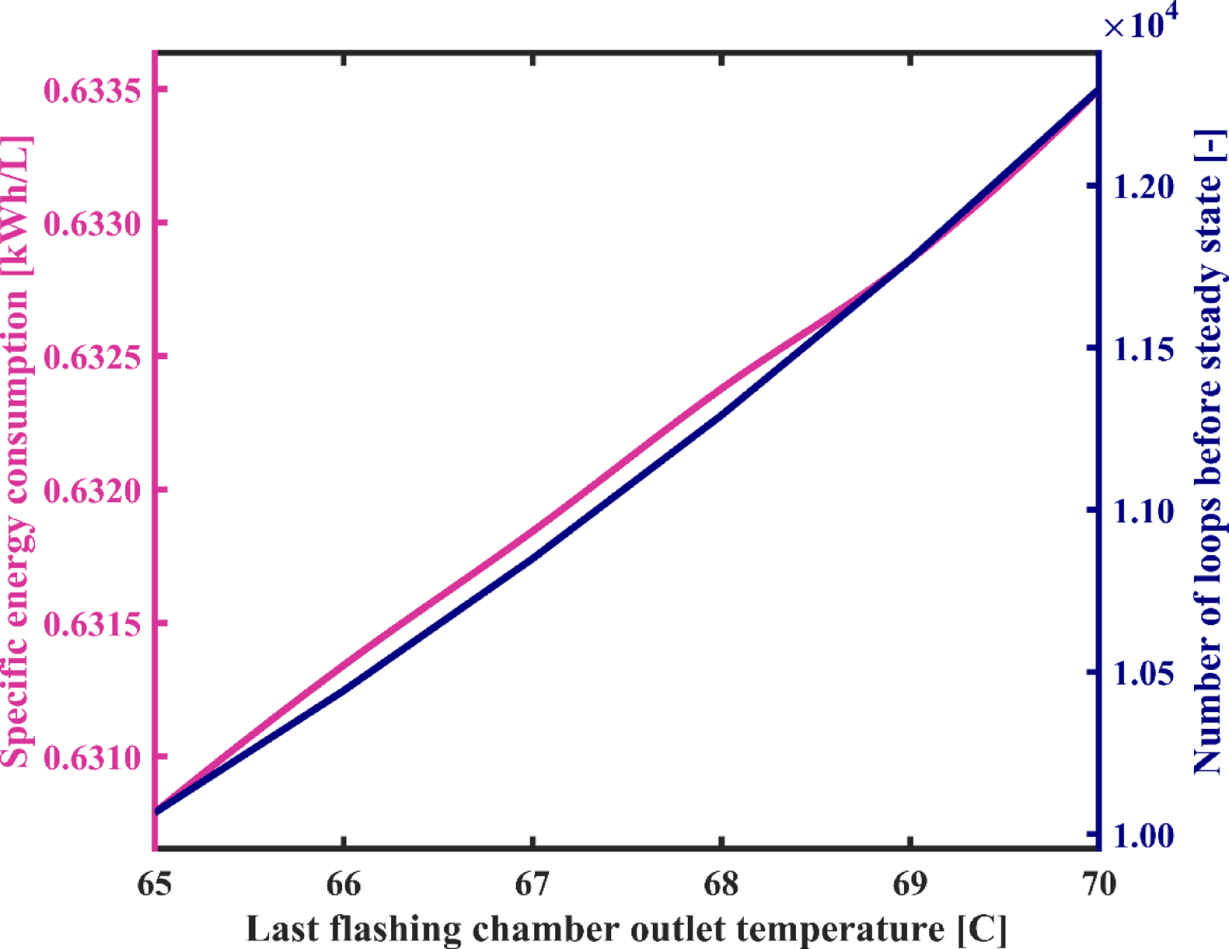
System’s specific energy consumption and number of water recirculation among condensation chambers before reaching steady state with varying flashing chambers outlet temperature.

As observed in Fig. 9, an increase in the flashing chambers outlet temperature ($\:{T}_{3}$) from 65 °C to 70 °C results in a decrease in the production flow rate of fresh water from 4.55 kg/h to 3.72 kg/h. This reduction occurs because with an increase in $\:{T}_{3}$, the enthalpy and energy of the brine exiting the flashing chambers increase (stream 3), while assuming a constant temperature for the heater ($\:{T}_{1}$), meaning the input energy to the system remains constant. Consequently, the difference between the input and output energies from the flashing chambers assembly decreases. This, in turn, implies a reduction in the output energy from the flashing chambers towards the condensation assembly, resulting in a decrease in the flashed vapor flow rate. Therefore, flow rate and temperature of stream 12, condensation chambers outlet, reduce, and its salinity increases. Consequently, the total rate of fresh water generation decrease. Furthermore, by increasing in the $\:{T}_{3}$, the temperature of the brine at the inlet of the heater increases. Since the temperature of the brine at the outlet of the heater is assumed to be constant, according to the energy balance equation for the heater in Table 1, the energy consumption of the heater decreases. Therefore, as it can be seen in Fig. 9, energy consumption of the desalination system reduces. On the other hand, the flow passing through the condensers takes longer to reach the desired salinity for fresh water due to the higher salinity of water at the condensation chambers outlet. As depicted in Fig. 10, where an increase in the $\:{T}_{3}$ from 65 °C to 70 °C leads to an increase in the number of loops required for water circulation before reaching steady state from 10,066 to 12,297. Also, changes in energy consumption per unit mass of fresh water produced are observed in Fig. 10. According to this figure, by increasing the flashing chambers outlet temperature, the SEC increases by 0.4%, the reason for which lies in the differing slopes of the reduction in energy consumption and the reduction in the flow rate of fresh water, as illustrated in Fig. 9. As depicted in Fig. 10, the SEC values remain approximately 0.63 kWh/L throughout the studied temperature range.

#### Brine tank outlet to distillate tank outlet mass flow rate ratio

**Fig. 11:**
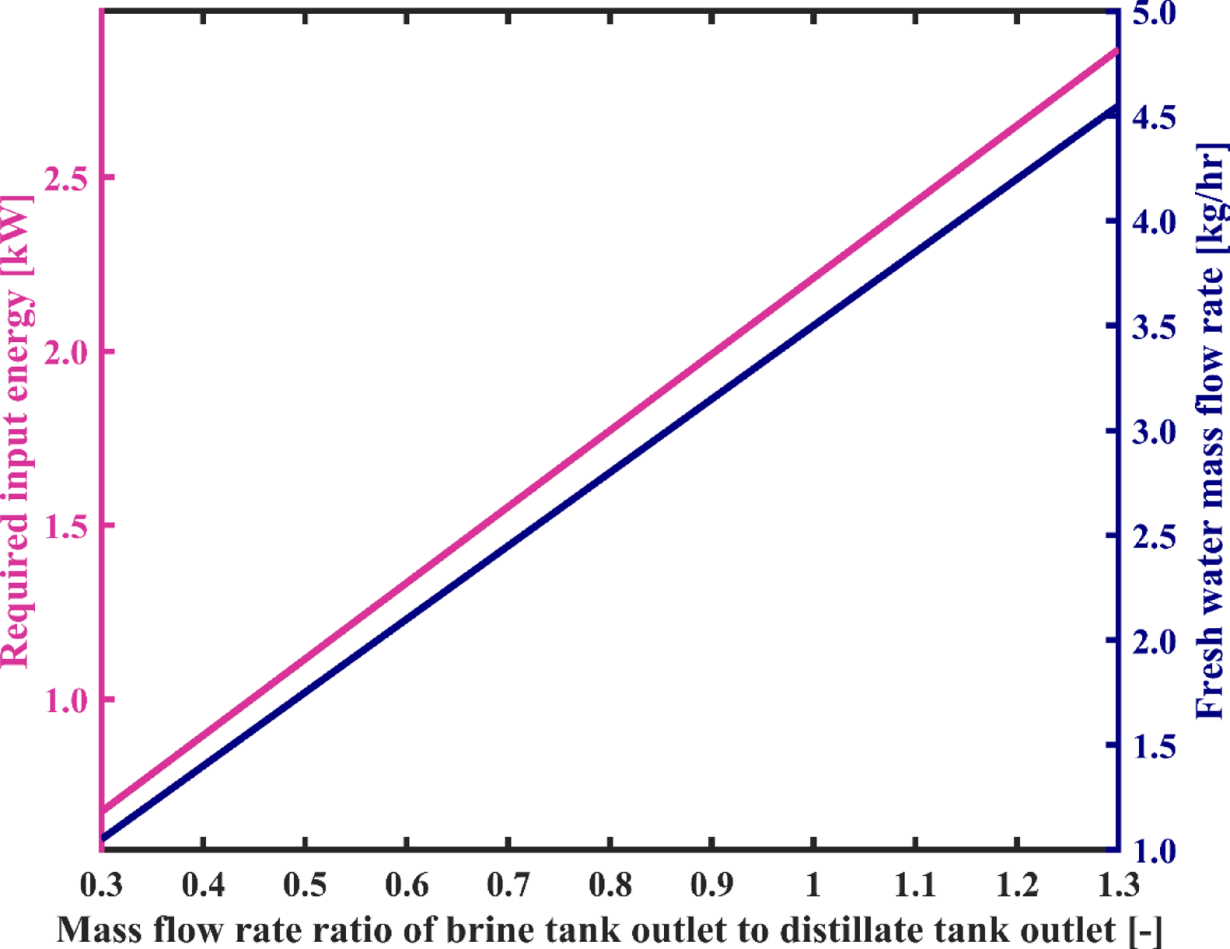
The variation of total required energy and fresh water production capacity as a function of mass flow rate ratio of brine tank outlet to distillate tank outlet.

In this section, the influence of the mass flow rate ratio of brine tank outlet to distillate tank outlet on the system performance is investigated. According to the results shown in Fig. 11, with a change in the flow rate ratio from 0.3 to 1.3, the system’s overall energy consumption rises from 0.68 kW to 2.87 kW. Additionally, the fresh water production capacity increases from 1.05 kg/h to 4.55 kg/h. This is due to the fact that raising the flow rate ratio, while considering the flow rate of the distillate tank outlet as constant, leads to an increase in the flow rate of the brine tank outlet. As a result, the energy required to heat it to the constant temperature of the heater also increases. On the other hand, despite the fact that the salinity of the input and output streams of the flashing chambers assembly increases and the enthalpy of these streams decreases, due to the increase in the flow rate in these two streams, the difference in brine energy between the input and output of the flashing assembly increases. Consequently, according to the total energy balance of the flashing chambers, more energy is transferred to the condenser chambers, and in fact, the flow rate of flashed vapor significantly increases. Therefore, the water stream passing through the condensation chambers receives a greater amount of vapor for condensation, resulting in higher flow rate and temperature at the output of the condensation chambers assembly, which in turn means an increase in the capacity of producing fresh water. On the other hand, the output flow rate of the flashing set increases, and consequently, the energy consumption by the brine pump also increases. Therefore, generally, with an increase in the flow rate ratio, the total energy consumption of the system experiences a significant increase.

**Fig. 12:**
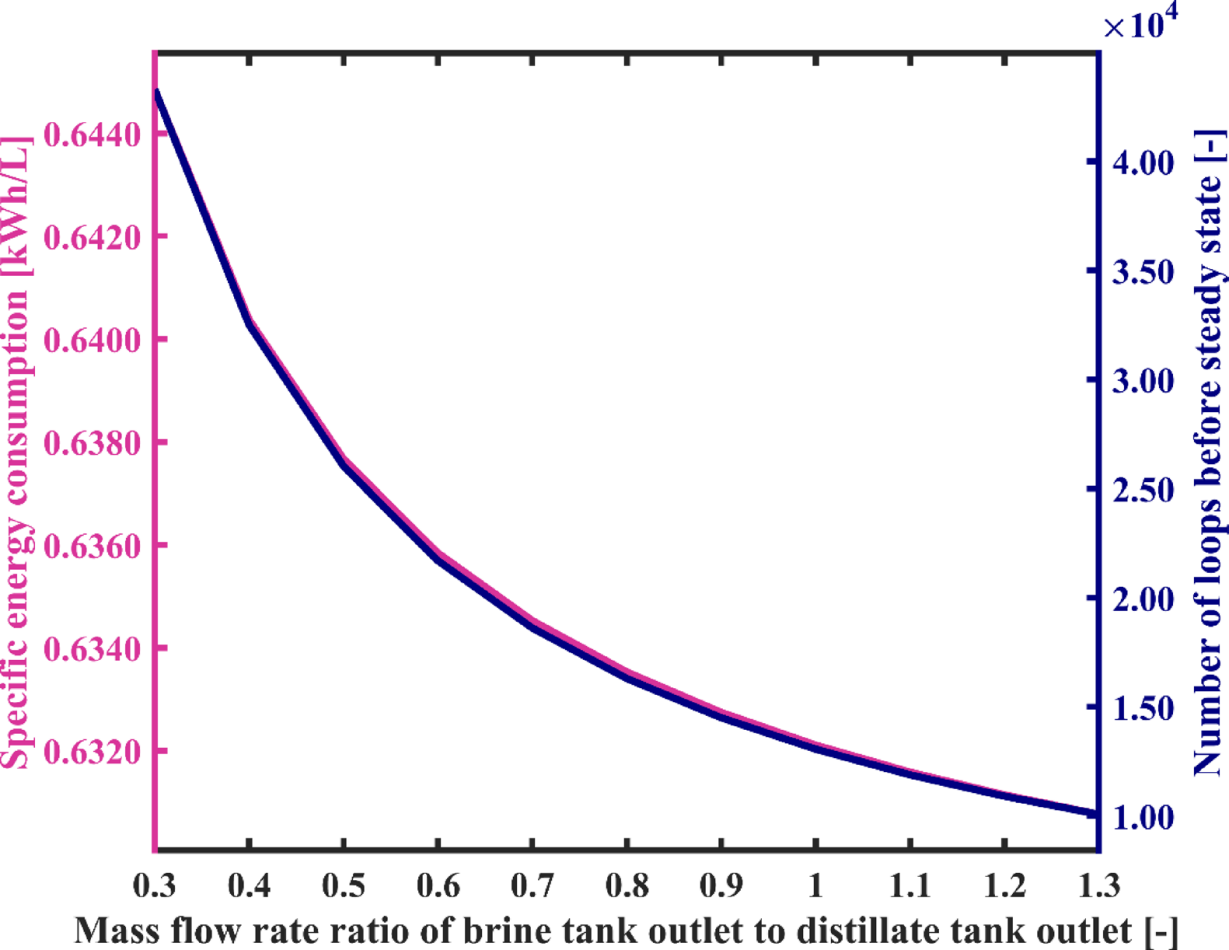
System’s specific energy consumption and number of water recirculation among condensation chambers before reaching steady state with varying mass flow rate ratio of brine tank outlet to distillate tank outlet.

Figure 12 illustrates the variation of number of loops during system startup as a function of the flow rate ratio. As mentioned earlier, the philosophy behind reducing the system startup duration also stems from this point, that with an increase in the amount of flashed vapor in each cycle of water circulation among the condensation chambers, water is sweetened more quickly and reaches the desired salinity for the product sooner. Therefore, the number of water circulation loops until reaching a steady operation decreases significantly. As observed in Fig. 12, the number of loops decreases from 43,297 to 10,069. In Fig. 12, changes in the SEC for different flow rate ratios are also observed. As seen in Fig. 11, both energy and the flow rate of fresh water exhibit an increasing trend. To determine the changes in the ratio of these two parameters, the slope of the changes of each individually becomes important. The results show that increasing the flow rate ratio from 0.3 to 1.3 decreases the SEC by 2.2%. Therefore, it would be more practical to adjust the flow rate ratio to a maximum value.

#### Feed water salinity

**Fig. 13:**
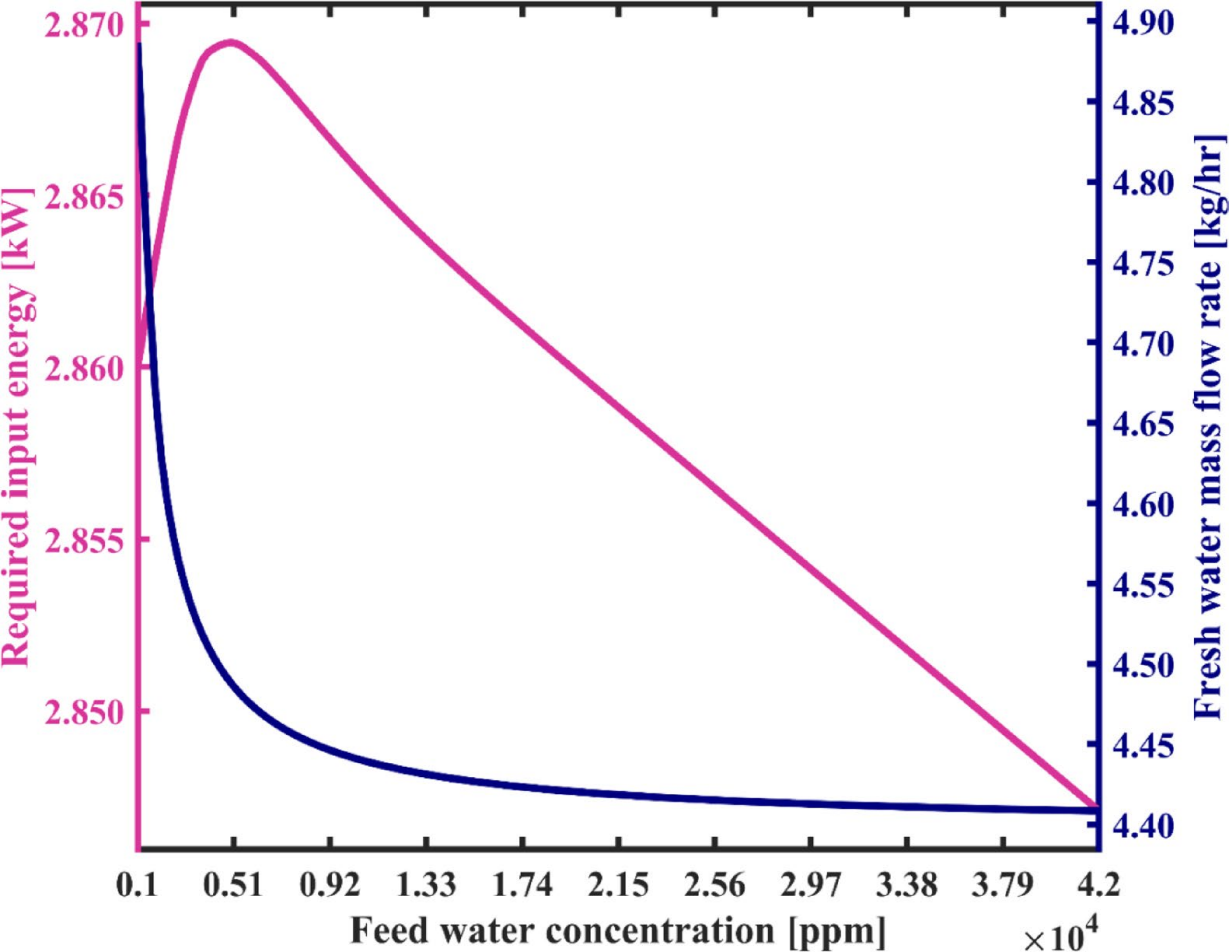
The total required energy and fresh water production capacity vs. the feed water salinity.

In this section the impact of feedwater salinity on the performance of the system under study state operation is investigated. The range of feedwater salinity is considered from 1000 ppm to 42,000 ppm, encompassing the widest possible range for salinity including brackish water and seawater salinity. It can be observed from Fig. 13 that with a rise in feedwater salinity from 1000 ppm to 5000 ppm, energy usage increases slightly, about 0.3%. For successive increases in the salinity up to 42,000 ppm, energy consumption continuously decreases with a total decline of about 0.8% compared to that at 5000 ppm. The changes are due to adjustments in feed and discharge flow rates to maintain mass and energy balances in the system. These affect the salinity and temperature—particularly at the brine tank outlet—which influences brine enthalpy according to the enthalpy correlation presented by Homig^41^ and slightly alters Heater energy demand. Furthermore, it is noted that the flow rate of fresh water also decreases slightly by 9.8%. In this figure, it is observed that with an increase in feedwater salinity from 1000 ppm to 9200 ppm, the reduction in the system’s fresh water production capacity occurs rapidly, and thereafter, with an increase in salinity up to 42,000 ppm, the reduction in the flow rate of fresh water becomes very insignificant. The reason for this reduction is that in the steady-state operation, with an increase in feedwater salinity entering the distillate tank, in order to adjust the salinity of the water within the distillate tank to achieve the appropriate salinity of product fresh water, the system is forced to increase the flow rate entering the fresh water reservoir so that the salinity inside the tank reaches an appropriate level for desalination during each water circulation, Hence, a smaller portion of stream 12 in Fig. 1(a) is extracted as fresh water from the system, and a larger portion of stream 12 returns to the distillate tank to balance the salinity within the reservoir tank according to the salt mass balance equation in the distillate tank.

**Fig. 14:**
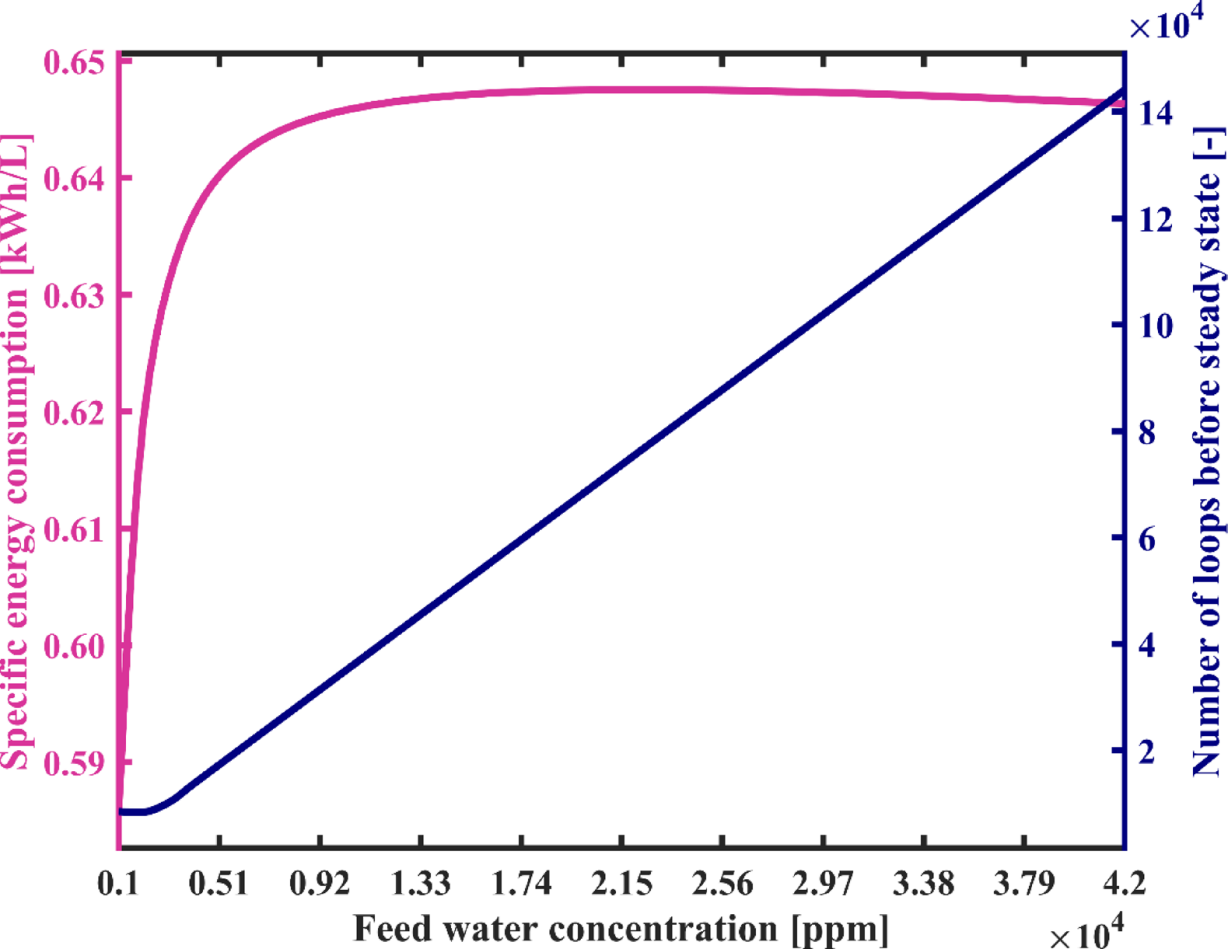
System’s specific energy consumption and number of water recirculation among condensation chambers before reaching steady state with varying feed water salinity.

In Fig. 14, variations in SEC and the number of water cycles in the system startup phase are observed concerning feedwater salinity. As the feed salinity increases from 1000 ppm to 5000 ppm, due to a slight rise in energy consumption and a significant drop in freshwater production—as shown in Fig. 13—a sharp increase of approximately 8.5% in SEC is observed. With further increase in feed salinity up to 42,000 ppm, the rate of SEC growth slows significantly, with only a 1% increase observed. Overall, with the increase in feedwater salinity, the SEC increases from 0.586 kWh/L to 0.646 kWh/L. This is due to the minor variations in both energy consumption and freshwater production, as illustrated in Fig. 13. Moreover, with an increase in feedwater salinity, the number of loops required for water circulation in the condensation chambers increases significantly. Therefore, the higher the initial salinity of the system’s feedwater, the longer and more extensive the startup process becomes. As shown in the Fig. 14, the number of loops required during startup increases significantly—from 8,512 to 144,150.

**Fig. 15:**
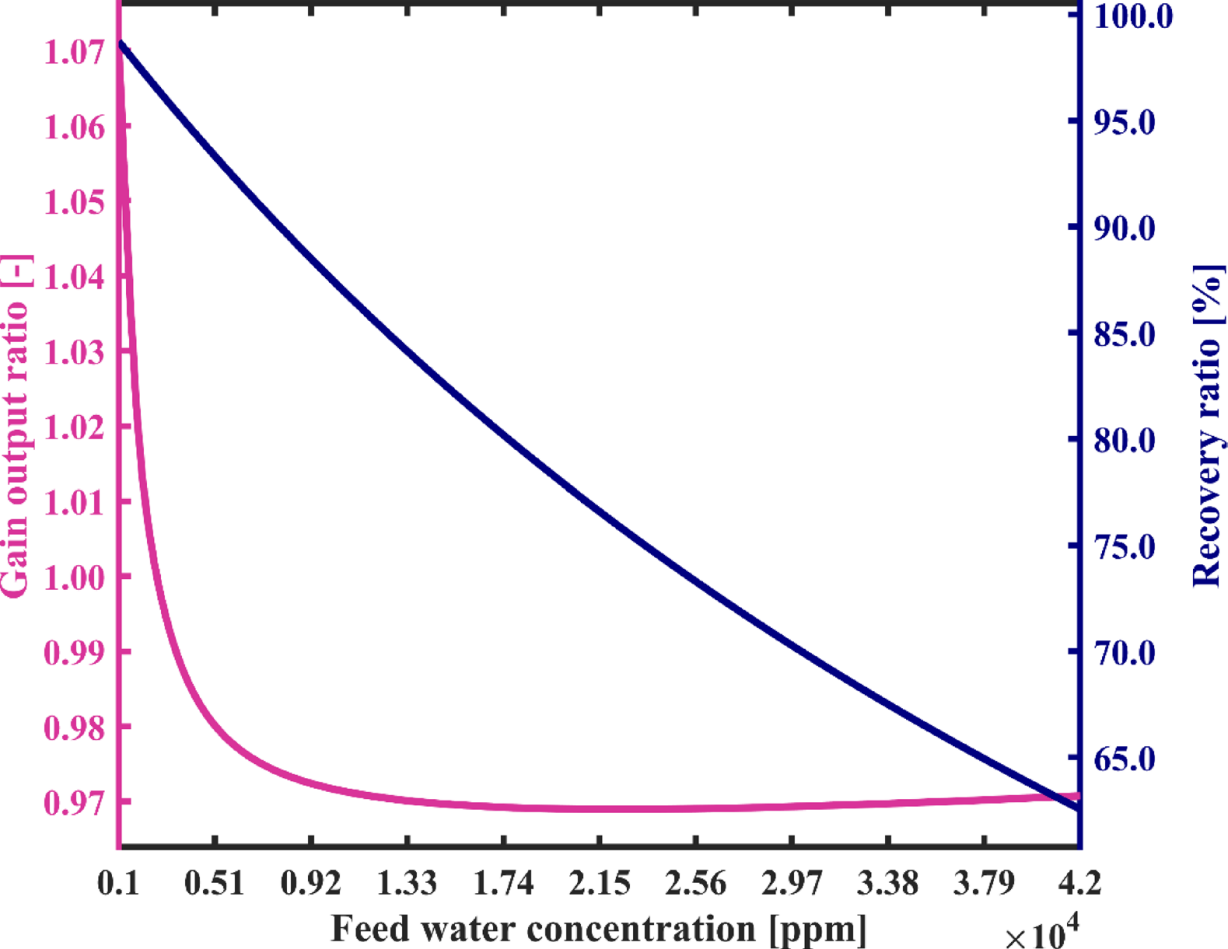
Gain output ratio and recovery ratio of the proposed system with varying feed water salinity.

Figure 15 shows the variations of recovery ratio (RR) and gain output ratio (GOR) against feed water salinity. According to the results illustrated in this figure, feed water salinity has the significant impact on the recovery ratio (RR) so that by increasing the feed water concentration from 1000 ppm to 42,000 ppm system’s recovery ratio is decreased from 98.73 to 62.55%. Due to variations in the salinity of the feedwater, all streams’ salinities change under steady-state conditions. Consequently, according to the salt mass balance equations of reservoir tanks, both the flow rates of the feedwater streams and the produced fresh water flow rate undergo changes. As a result, recovery ratio also varies according to Eq. (24). Therefore, the proposed desalination system can effectively desalinate seawater and especially brackish water with a considerable recovery ratio. Due to the absence of heat transfer surfaces inside the chambers, concerns about scale formation are minimal. As a result, the salinity of the discharged brine from the system can be significantly increased, and a minimal liquid discharge approach can be obtained, providing distinct environmental benefits. Moreover, according to Eq. (25), the GOR is proportional to the ratio of the mass of produced freshwater to the heat transferred in the heater. Since the heater accounts for the majority of the system’s energy input, the GOR can be considered approximately inversely proportional to the SEC. Therefore, its trend in Fig. 15 is observed to be decreasing, in contrast to the increasing trend of the SEC. Consistently, the GOR decreases from 1.07 to 0.97 as the feedwater salinity increases.

#### The maximum allowable salinity for produced fresh water

The maximum permissible salinity of treated freshwater shall be determined taking into account the intended use of the proposed freshwater e.g., if freshwater is intended for agricultural and greenhouse use, the maximum allowed salinity will vary depending on the type of product being grown. In this study, the effect of the maximum permissible concentration of 100 ppm, suitable for drinking, up to 1000 ppm, covering a wide range of concentrations suitable for different applications, has been studied on the system.

**Fig. 16:**
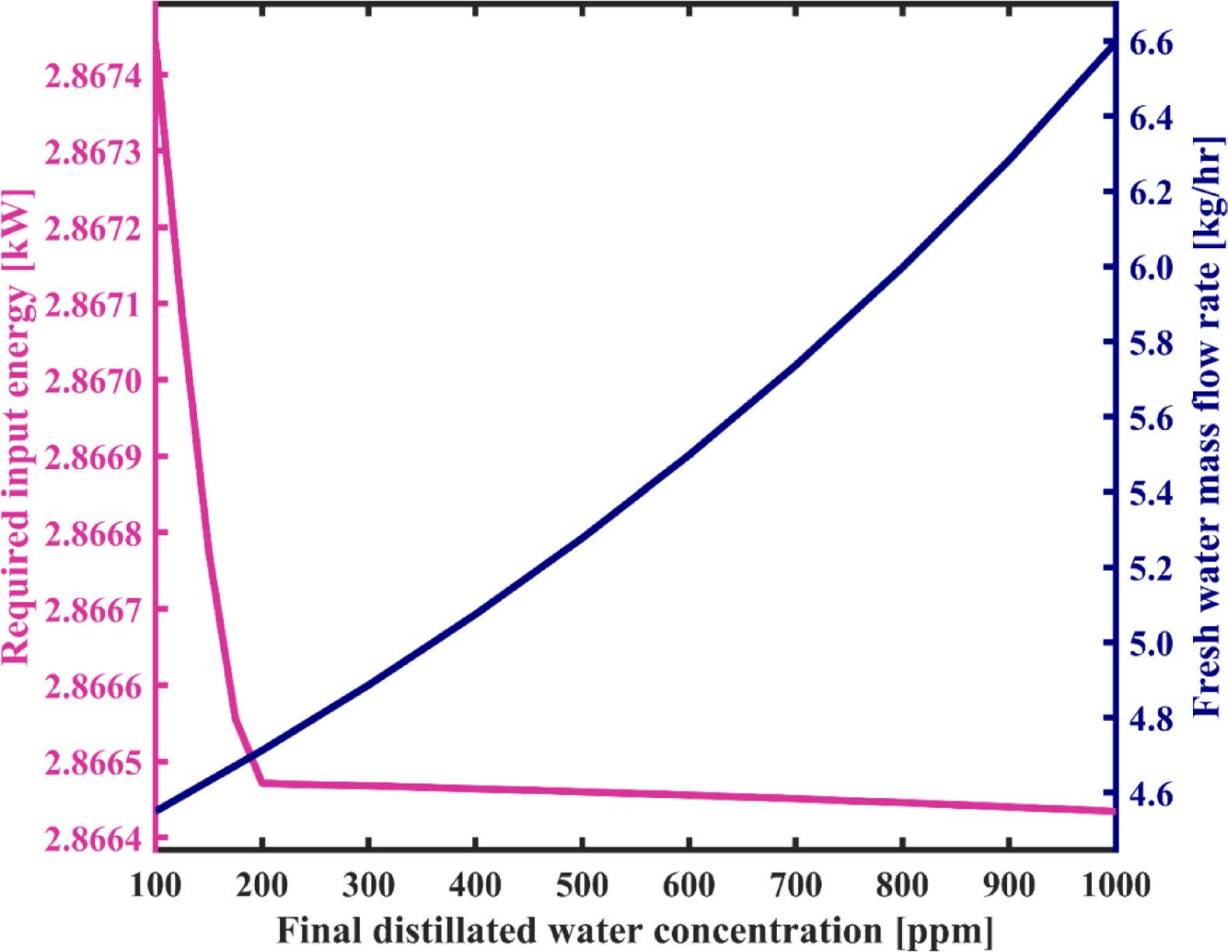
The variation of total required energy and fresh water production capacity as a function of final distillated water concentration.

It can be seen from Fig. 16 that increasing the permissible salt concentration in the produced freshwater from 100 ppm to 1000 ppm leads to a negligible decrease in energy consumption—approximately 0.03%. The reason for this slight decrease is that only streams salinity changes by adjusting the maximum freshwater concentration under steady-state operating condition. According to the salt water enthalpy function^41^ which is a function of temperature and salinity, the enthalpy changes result in a small change in the energy gained in the brine Heater. Therefore, the total energy change will be small. Furthermore, according to this figure, an increase in the salinity of the product increases the fresh water flow rate from 4.55 kg/h to 6.6 kg/h, which is a considerable variation. This variation is due to the fact that the concentration of all streams changes during steady-state operating conditions as the maximum allowable salinity of the product increases. Therefore, in order to balance the salt in the tanks, the flow rates should be adjusted.

**Fig. 17:**
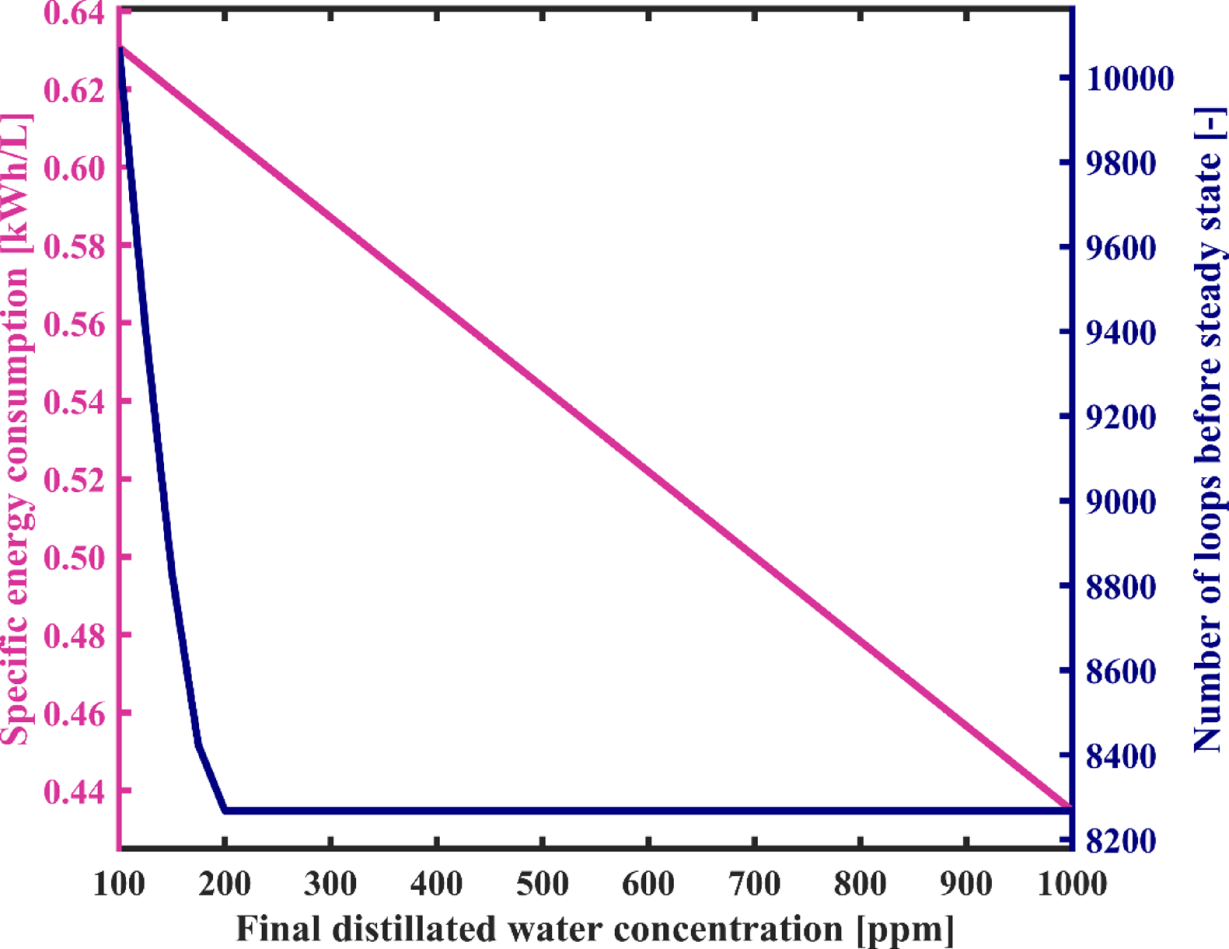
System’s specific energy consumption and number of water recirculation among condensation chambers before reaching steady state with varying final distillated water concentration.

**Fig. 18:**
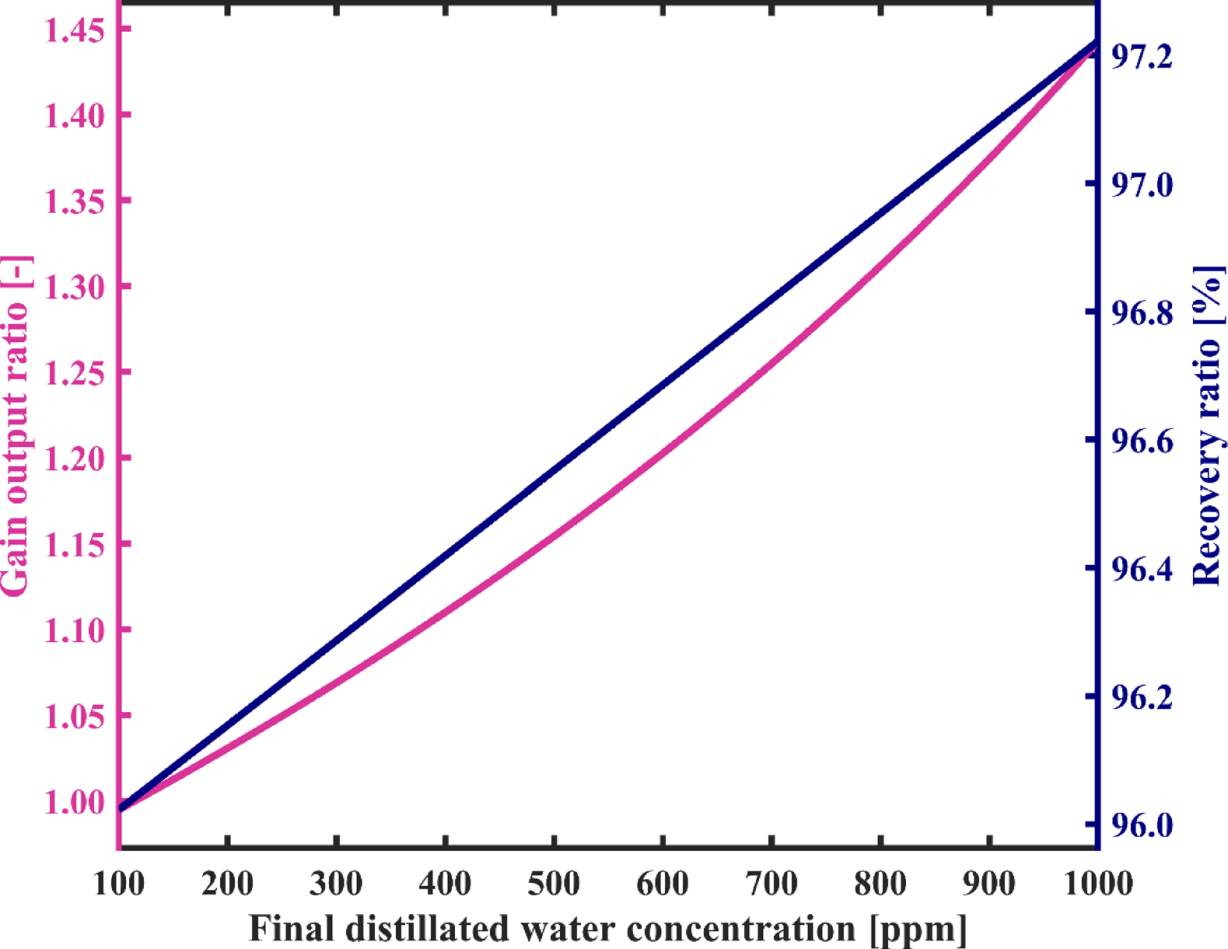
Gain output ratio and recovery ratio of the proposed system as a function of final distillated water concentration.

According to the results shown in Fig. 17, it is clear that an increase in the maximum allowable salinity of produced fresh water decreases the number of loops required for water circulation in the condensation chambers. This is because the difference between the salinity of the feedwater initially fed into the distillate tank and the salinity of the fresh water decreases. It can be seen from this figure that the number of circulation loops decreases sharply from 10,071 to 8268 as the maximum allowable concentration of the product increases from 100 ppm to 200 ppm, and its value gradually decreases with the salinity of the product rise up to 1000 ppm. Figure 17 shows that the SEC decreases by 31% as freshwater quality decreases, which is influenced by the curves depicted in Fig. 16. According to Fig. 17, SEC decreases from 0.631 to 0.435 kWh/L. Therefore, the GOR also exhibits an increasing trend, as observed in Fig. 18, rising from 0.99 to 1.44. In addition, as mentioned earlier, the recovery ratio shows a slight improvement of 1.2% as a result of adjusting the feedwater and freshwater flow rates in response to the increase in the final salinity of the distillate.

### Preliminary economic evaluation

To evaluate the economic feasibility of the proposed desalination system, a simplified life-cycle cost analysis is conducted to estimate the levelized cost of freshwater production (LCOW). Because the system will be installed in off-grid applications in rural regions, the research concerns solar-driven operation without any auxiliary energy supply. As explained in the previous sections, the pilot city involved in this research is Chabahar, which is a southeast coastal city in Iran with great solar irradiation. This region receives approximately 3072 h of sunshine annually and has an average daily solar irradiation of 6.5 kWh/m²/day^38^.The system is assumed to operate solely during sunny hours over 360 days per year. Based on the annual sunshine hours in Chabahar, it is assumed that the system operates for 8 h per day. Considering the freshwater production rate of 4.7 L/h, the total annual freshwater yield of the proposed desalination system is estimated to be approximately 13.54 m³. Given the previously calculated specific thermal energy consumption of 626 kWh/m³, the total annual thermal energy demand for freshwater production can be determined. Moreover, based on the average solar irradiation in Chabahar and assuming an efficiency of 60% for the evacuated tube solar collectors, the useful thermal energy received per square meter is obtained. Dividing the annual thermal energy requirement by the useful energy per unit collector area results in a required solar collector surface area of approximately 6 m². A similar approach is applied to the system’s electrical energy demand. Based on that, a 100 W photovoltaic panel is sufficient to power the three desalination unit’s pumps and one solar collector circulation pump, with a safety margin of over 45% in daily energy production.

Table 6 presents the capital costs of major components, including desalination unit, vacuum tube solar collectors, circulation pumps and photovoltaic module. Then, the total capital expenditure (CAPEX) is estimated and annualized CAPEX is calculated according to the equation in the Table 6. In this study the annual operational expenditure (OPEX) is taken as 5% of CAPEX, and the interest rate is assumed to be 4%^47^. In addition, with appropriate maintenance, the system lifetime is projected to be 20 years. The capital recovery factor (CRF) is calculated based on the following equation:

26 $$CRF=\frac{{i{{(1+i)}^n}}}{{{{(1+i)}^n} - 1}}$$

Where *i* is the interest rate and *n* is the life of the system (in years). Finally, the levelized cost of water (LCOW) is estimated by dividing the total annual cost by the annual freshwater production, resulting in approximately 13.78 US$/m³ (0.014 US$/L).

Recent studies confirm that the LCOW for small-scale solar desalination systems typically ranges from approximately 10 to 84 USD/m³, depending on system complexity, use of tracking technologies, and production capacity. For example, solar stills without tracking systems operate in the range of 12–28 US$/m³^48^, while solar HDH systems achieve 10–18 US$/m³^49^. The proposed small scale desalination system, with an estimated cost of 13.78 US$/m³, clearly falls within these ranges, supporting its economic viability for deployment in remote and off-grid areas.

**Table 6:** The cost of desalination unit with evacuated tube solar collector^47^.

Item	Unit	Quantity	Unit cost (US$)	Total cost (US$)
Evacuated tube solar collector	m^2^	6	150	900
Desalination system (UPVC pipes and tanks- insulation-polyethylene pipes of GHE)	-	1	200	200
Circulation pumps (desalination unit’s pumps and collectors pump)	-	4	60	240
100 W solar panel	-	1	70	70
Labor works	-	1	100	100
Capital Expenditures (CAPEX)	-	-	-	1510
Annualized CAPEX = $\:CRF\times\:CAPEX$	-	-	-	111.14
Operational Expenditures (OPEX) = *0.05*$\:\times\:CAPEX$	-	-	-	75.5
Total Annual Cost=$\:OPEX+Annulized\:CAPEX$	-	-	-	186.64

### Performance comparison with existing desalination technologies

Although the proposed system is technically inspired by the Multi-Stage Flash (MSF) process—utilizing similar flashing and condensation mechanisms—it differs fundamentally from both conventional MSF and reverse osmosis (RO) in terms of scale, energy source, and intended application. A detailed technical comparison with these industrial benchmarks is provided in Table 7. Conventional MSF plants are designed for large capacities^50^ (> 4,000 m³/day) where economies of scale justify their high capital and operational complexity, making them economically unviable for small-scale applications due to their large physical footprint and rigid structure^51,52^. Furthermore, key technical barriers prevent their adaptation to small-scale or low-temperature operation, despite the fact that the proposed system only overlaps with MSF in the narrow top brine temperature range of 90–95 °C.

- High-grade heat dependency: Conventional MSF requires stable and high-pressure steam (2–3 bar) by using steam usually spilled from a power plant not only for brine heating but primarily to drive thermal vapor compressors (TVCs), which are essential for achieving a high GOR. This necessitates a high-grade thermal infrastructure^53,54^.
- Deep vacuum requirement: MSF relies on a deep vacuum in final stages (brine outlet temperature of approximately 40 °C)^40,46^ for efficiency, requiring complex sealing and maintenance.

In contrast, the proposed system operates at a higher final-stage temperature (> 65 °C), reducing the vacuum requirement and simplifying maintenance. It eliminates TVCs and internal heat transfer tubes, removing the need for high-pressure steam and drastically reducing scaling risk. By forgoing high GOR for operational simplicity, the system becomes compatible with low-grade heat and uniquely viable for remote, off-grid applications. In addition, by continuously recirculating brine and freshwater through pumps and storage tanks, the system—unlike MSF—achieves a much higher recovery ratio (up to 96%), which is a critical advantage for remote areas with limited saline water resources as it maximizes freshwater yield from scarce feedwater. Table 7 summarizes these trade-offs.

**Table 7:** Technical comparison of the proposed system with MSF and RO systems.

Feature	Conventional MSF	Industrial RO	Proposed Loop-Configured MSF system
Scale	Large-scale (e.g., > 4,000 m³/day)^50^	Medium to large-scale (e.g., > 1200 m³/day)^55^	Small-scale (0.1 m³/day)
Desalination Mechanism	Thermal (flashing/condensation)	Membrane separation (pressure-driven)	Thermal (flashing/condensation)
Top Brine Temperature (TBT)	90–120°C^56^	Ambient (with pressurization)	< 95 °C (Below boiling point)
Last-stage brine temperature	~ 40 °C (Higher vacuum percentage, significantly lower SEC)	Not applicable	> 65 °C (Lower vacuum requirement and higher SEC, easier sealing & maintenance)
Energy Source	High-grade steam or thermal	High-pressure pumps (electric)	Low-grade thermal (solar/geothermal)
Specific Energy Consumption (SEC)	55–80 kWh/m³ thermal^57^ 2.5–3.5 kWh/m³ electrical^58^	3.7–8 kWh/m³ electrical^59^	626 kWh/m³ thermal 4.8 kWh/m³ electrical
GOR	6.5–8^60^	Not applicable	1
Recovery Ratio	20–35%^61^	35–45% typical^62^	> 96% (Minimum Liquid Discharge)
Fouling/Scaling Risk	High (requires frequent cleaning)	Membrane fouling, scaling	Low (no internal tube surfaces, no membrane)
Maintenance Complexity	High (Scaling, fouling, corrosion, steam ejector, frequent chemical cleaning, trained operators)	Medium–high (membrane replacement, post/pre-treatment, pressurization)	Low (simple looped design, no ejectors, no internal tubes and low scaling risk), suitable for off-grid use
Suitability for Remote Areas	Poor	Moderate (with renewable-electric support)	High

It is important to note that direct comparison with MSF and RO methods based on energy performance metrics such as SEC or GOR is not meaningful due to the vastly different operational contexts. The proposed system is designed for small-scale, low-maintenance, off-grid use, making it more appropriate to benchmark its performance against other decentralized desalination technologies. Accordingly, Table 8 presents a comparative evaluation with small-scale systems such as HDH, MD, and thermoelectric desalination units. As shown in Table 8, the proposed system demonstrates a unique combination of high recovery ratio (96%) and low fouling risk, which is rarely achieved in other decentralized desalination technologies. In addition, quantitative comparisons reveal significant performance improvements. The proposed system achieves a 9.1% higher RR and a 53.8% higher GOR than the HDH system by Dehghani et al.^63^. Another HDH system studied by Rajaseenivasan & Srithar^64^ exhibits a GOR similar to that of the proposed system. More strikingly, the proposed system outperforms the thermoelectric (TE) system by Al-Madhhachi & Min^65^ by a remarkable 81.8% in GOR while consuming 44.7% less specific energy (SEC), and delivers a water recovery ratio more than ten times higher. According to Table 8, the GOR value of the studied desalination system (1.0) is at the lower bound of the range reported for the studied MD systems by Zuo et al.^66^ and Mohamed et al.^67^. Although the vacuum membrane distillation (VMD) system integrated with a crystallizer, investigated by Zuo et al.^66^ demonstrates a RR nearly identical to that of the proposed desalination system (95%), the proposed system achieves a 13–92% higher RR compared to the vacuum multi-effect MD system reported by Mohamed et al.^67^ across its operating range. Moreover, the SEC of the proposed system (0.63 kWh/L) falls within the performance range of this vacuum multi-effect MD system (0.3–0.7 kWh/L)^67^. This competitive energy consumption, combined with a higher recovery ratio (96% vs. 50–85%) and the elimination of membrane fouling concerns, highlights its practical advantages for remote applications. Unlike HDH systems, which require precise control of heat and mass flows, and TE systems with low efficiency and limited scalability, the proposed looped thermal design relies on simple flashing and condensation without membranes or complex modules. These features, along with its competitive levelized cost of water (LCOW), make it easier to construct, operate, and maintain in remote settings.

**Table 8:** Comparison of performance metrics between the proposed system and other decentralized desalination technologies.

Author(s)	Studied system	SEC (kWh/L)	Water Production	Recovery ratio (%)	GOR	Remarks
Present work	A novel small scale desalination system with loop-configured MSF and reservoir tanks	0.63	4.7 (L/h)	96	1	Both thermal and electrical energy were included in SEC.
1-Humidification–Dehumidification Desalination (HDH)						
Dehghani et al.^63^	Open-air, open-water HDH system	-	4.9 (L/h)	88	0.65	- A gas burner powered the cycle. - The recirculation of brine was investigated.
Rajaseenivasan & Srithar^64^	Open-air, open-water HDH system	**-**	6.1 (L/h)	-	1	-Biomass fuel was used to power the cycle.
2- Thermoelectric Desalination (TE)						
Al-Madhhachi & Min^65^	thermoelectric (TE) desalinator	1.14	0.684(L/m^2^/h)	9.5	0.55	-This system relies only on electricity. -Fresh water production is based on condenser area.
3-Membrane Distillation (MD)						
Zuo et al^66^.	Vacuum membrane distillation (VMD) integrated with a crystallizer for zero-brine discharge	**-**	3.7 (L/m^2^/h)	95	1-1.59	-The increase in GOR is due to the recovery of heat lost from the condenser. -Brine recirculation is employed to enhance the recovery ratio.
Mohamed et al^67^.	Vacuum multi-effect membrane distillation (V-MEMD)	0.3–0.7	2.58–7.71 (L/m^2^/h)	50–85	1-2.2	-

## Conclusion

In this paper, a novel small scale desalination system was proposed. This system is a loop-configured MSF system with reservoir tanks, where simplicity of operation, structure, and maintenance have been taken into consideration to the extent possible, making it attractive for desalinating water sources in remote areas. The proposed desalination system consists of sequential flashing and condensation chambers and operates based on two phenomena: flashing and vapor condensation. Vapor condensation in the distillation chambers occurs due to direct contact with the lower temperature saline water surface without using any tube. Therefore, the sensitivity of this system to high salinity water and scale formation is reduced. Further, the loop configuration of the system permits the internal recycling of the brine such that the salinity of the brine tank is gradually increases up to at least 70,000 ppm before continuous brine discharge begins. This allows the system to achieve a high-water recovery ratio of around 96% with minimal wastage of the liquid. As well as that, using the reservoir tanks in the system provides the capability to store the produced freshwater and brine. The system is capable of utilizing low-grade heat sources such as solar energy and relies on passive cooling to produce freshwater. In this study, for this novel system, thermodynamic assessment was conducted and the impact of key decision variables and parameters on the system efficiency was examined through an extensive parametric study. Additionally, a genetic algorithm-based multi-objective optimization was carried out to demonstrate optimal results. According to this optimization, Pareto Frontier of the studied system was presented, and the optimal range for each decision variable was shown by providing scatters of distribution for the variables. The most significant achievements of this paper can be expressed as:

- At the optimal solution (Point B on the Pareto frontier) under the following key operating conditions—feedwater salinity of 3000 ppm, feedwater temperature of 30 °C, heater outlet temperature ($$\:{T}_{1}$$) of 91.5 °C, flashing chamber outlet temperature ($$\:{T}_{3}$$) of 65 °C, and a mass flow rate ratio ($$\:{\dot{m}}_{6}$$/$$\:{\dot{m}}_{9}$$) of 1.2998—the system produced 4.7 L/h of freshwater. This was achieved with a specific energy consumption (SEC) of 0.6307 kWh/L and a gain output ratio (GOR) of 1. In this operating point, the system operated at a pressure range of 25–69 kPa across the flashing chambers. Under these conditions, the system achieved a recovery ratio of 96% and followed a minimum liquid discharge approach, which is highly valuable for desalinating limited saline water resources in an environmentally friendly manner.
- Decision variable scatter plots showed that the ideal range for decision variables was as follows: flashing chambers outlet temperature ($$\:{T}_{3}$$) = 65 °C (at the lower bound of the defined range), heater temperature ($$\:{T}_{1}$$) = 88 °C to 95 °C (upper portion of the bounds), mass flow rate ratio of brine tank outlet to distillate tank outlet ($$\:{\dot{m}}_{6}$$/$$\:{\dot{m}}_{9}$$) = 1.3 (at the upper bound of the defined range). Furthermore, within this optimal heater temperature range (88–95 °C), the desalination capacity varies from 3.96 to 5.04 L/h.
- The outcomes of the parametric analysis indicated that the outlet flow rate ratio of the two tanks had the strongest influence on system performance, causing up to 76–77% variation in energy use, freshwater production, and system startup duration, while reducing SEC by only 2.2%. In addition, the salinity of the feedwater and the desired salinity of the fresh water depending on the intended application strongly influenced the performance of the system. Thus, by varying the desired salinity of produced fresh water from 100ppm to 1000ppm, the SEC was reduced by 31%.
- The proposed system was benchmarked against various small-scale desalination technologies such as humidification–dehumidification (HDH), membrane distillation (MD), solar stills, and thermoelectric (TE) systems. It demonstrated competitive or superior performance in terms of water recovery, low fouling risk, and a competitive levelized cost of water (LCOW ≈ 0.014 US$/L). Moreover, its simple looped configuration and minimal maintenance requirements for off-grid use further underscore its practicality and operational ease, making it a promising solution for freshwater supply in remote and resource-limited settings.

## Data Availability

The data used in this study are available upon reasonable request from the corresponding author.
